# Supplementary material for: Comparative analysis of predicted DNA secondary structures infers complex human centromere topology
Source: Am J Hum Genet. 2024 Nov 18;111(12):2707–19. doi: 10.1016/j.ajhg.2024.10.016 (PMC11639080; doi:10.1016/j.ajhg.2024.10.016)
Supplement: Document S1. Figures S1–S6 [file mmc1.pdf]

The American Journal of Human Genetics, Volume *111*

Supplemental information

Comparative analysis of predicted DNA secondary structures infers complex human centromere topology

Sai Swaroop Chittoor and Simona Giunta

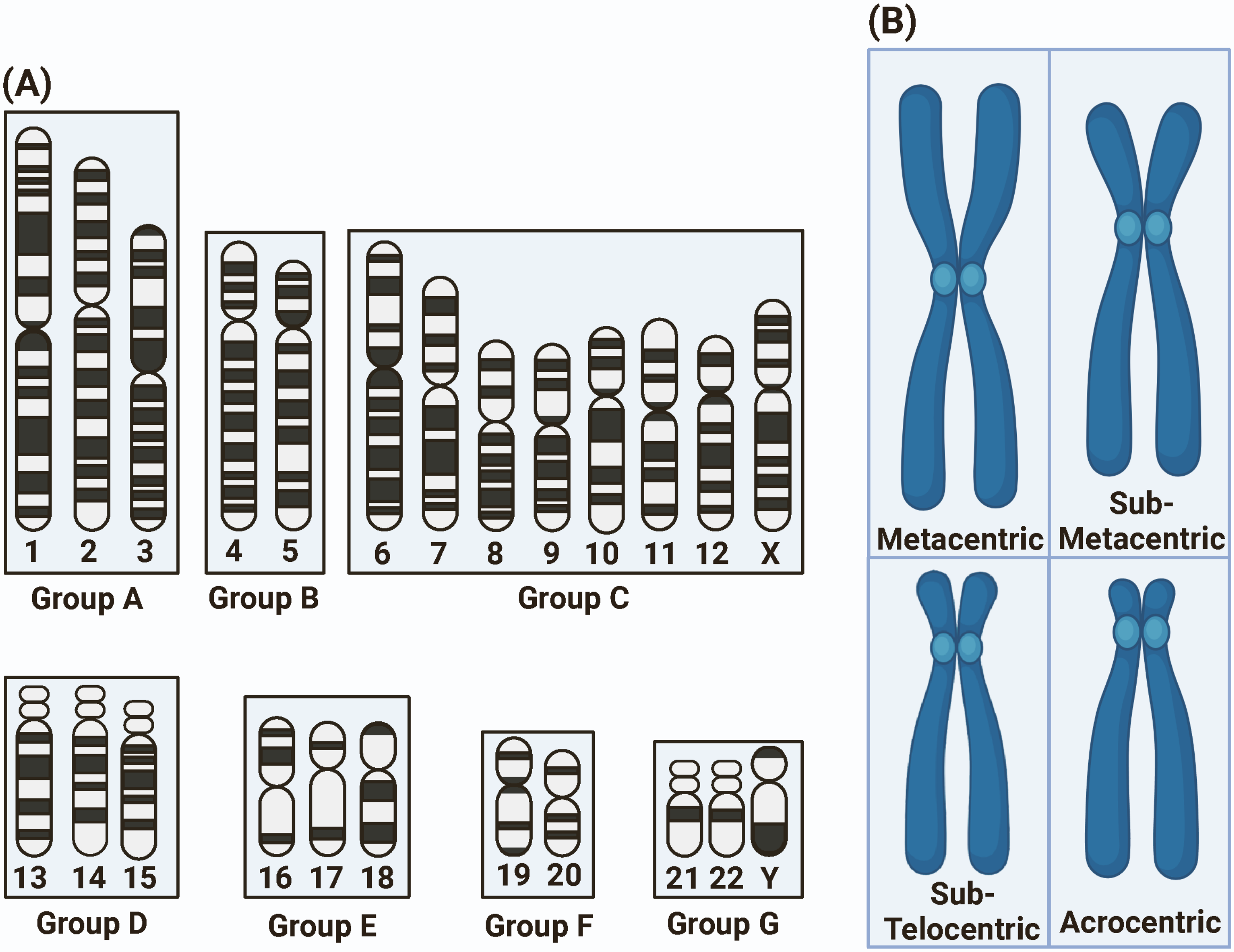
(A)
1
2
3
Group A
4
5
Group B
6
7
8
9
10
11
12
X
Group C
13
14
15
Group D
16
17
18
Group E
19
20
Group F
21
22
Y
Group G
(B)
Metacentric
Sub-
Metacentric
Sub-
Telocentric
Acrocentric

FIGURE S1

Figure S1. Classification of chromosomes based on centromere position. (A) Group-wise classification of 22+2 chromosomes in the human karyotype. Group A contains chromosomes 1, 2 and 3. These chromosomes are the largest and are metacentric with a centrally positioned centromere with two equivalent arms. Group B consists of chromosomes 4 and 5 which are submetacentric. Group C is the one with the largest number of chromosomes. It consists of chromosomes 6-12 and chromosome X. Group D comprises acrocentric chromosomes - 13,14 and 15. Group E contains chromosomes 16, 17 and 18, which are short meta/submetacentric chromosomes. Group F consists of short metacentric chromosomes 19 and 20 and finally Group G comprises very short acrocentric chromosomes 21, 22 and chromosome Y. (B) Four distinct types of chromosomes based on the centromere position. The figure was created using BioRender (2020).

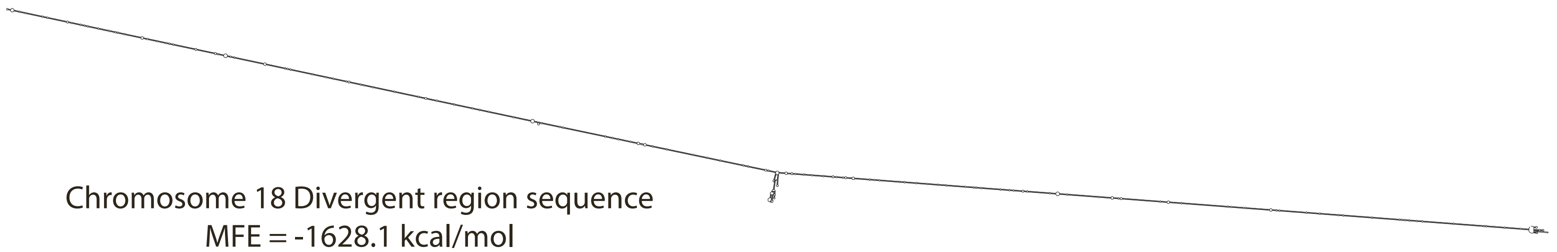

Chromosome 18 Divergent region sequence
MFE = -1628.1 kcal/mol

FIGURE S2

Figure S2. Secondary structure prediction of a DNA sequence from the divergent region of chromosome 18. Despite the sequence belonging to the divergent region, this secondary structure is the most stable with the lowest MFE value.

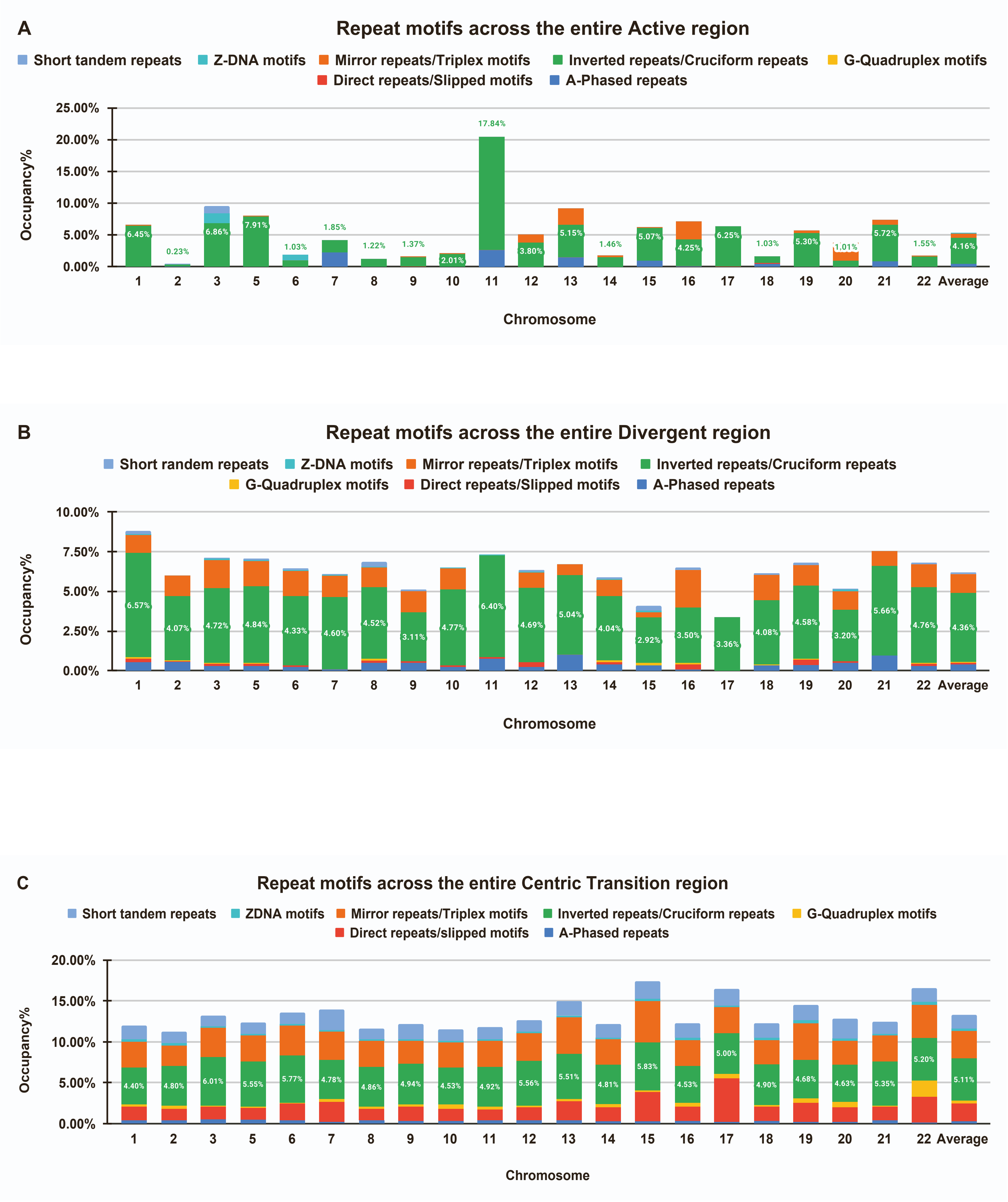
A
Repeat motifs across the entire Active region
Short tandem repeats
Z-DNA motifs
Mirror repeats/Triplex motifs
Inverted repeats/Cruciform repeats
G-Quadruplex motifs
Direct repeats/Slipped motifs
A-Phased repeats
Occupancy%
25.00%
20.00%
15.00%
10.00%
5.00%
0.00%
6.45%
0.23%
6.86%
7.91%
1.03%
1.85%
1.22%
1.37%
2.01%
17.84%
3.80%
5.15%
1.46%
5.07%
4.25%
6.25%
1.03%
5.30%
1.01%
5.72%
1.55%
4.16%
1 2 3 5 6 7 8 9 10 11 12 13 14 15 16 17 18 19 20 21 22 Average
Chromosome
B
Repeat motifs across the entire Divergent region
Short randem repeats
Z-DNA motifs
Mirror repeats/Triplex motifs
Inverted repeats/Cruciform repeats
G-Quadruplex motifs
Direct repeats/Slipped motifs
A-Phased repeats
Occupancy%
10.00%
7.50%
5.00%
2.50%
0.00%
6.57%
4.07%
4.72%
4.84%
4.33%
4.60%
4.52%
3.11%
4.77%
6.40%
4.69%
5.04%
4.04%
2.92%
3.50%
3.36%
4.08%
4.58%
3.20%
5.66%
4.76%
4.36%
1 2 3 5 6 7 8 9 10 11 12 13 14 15 16 17 18 19 20 21 22 Average
Chromosome
C
Repeat motifs across the entire Centric Transition region
Short tandem repeats
ZDNA motifs
Mirror repeats/Triplex motifs
Inverted repeats/Cruciform repeats
G-Quadruplex motifs
Direct repeats/slipped motifs
A-Phased repeats
Occupancy%
20.00%
15.00%
10.00%
5.00%
0.00%
4.40%
4.80%
6.01%
5.55%
5.77%
4.78%
4.86%
4.94%
4.53%
4.92%
5.56%
5.51%
4.81%
5.83%
4.53%
5.00%
4.90%
4.68%
4.63%
5.35%
5.20%
5.11%
1 2 3 5 6 7 8 9 10 11 12 13 14 15 16 17 18 19 20 21 22 Average
Chromosome

FIGURE S3

Figure S3. Repeat motif occupancy. Occupancy% of short tandem repeats, Z-DNA motifs, mirror repeats, inverted repeats, G4 motifs, Direct repeats and A-Phased repeats in the entirety of the (A) Active region, (B) Divergent region and (C) Centric Transition region.

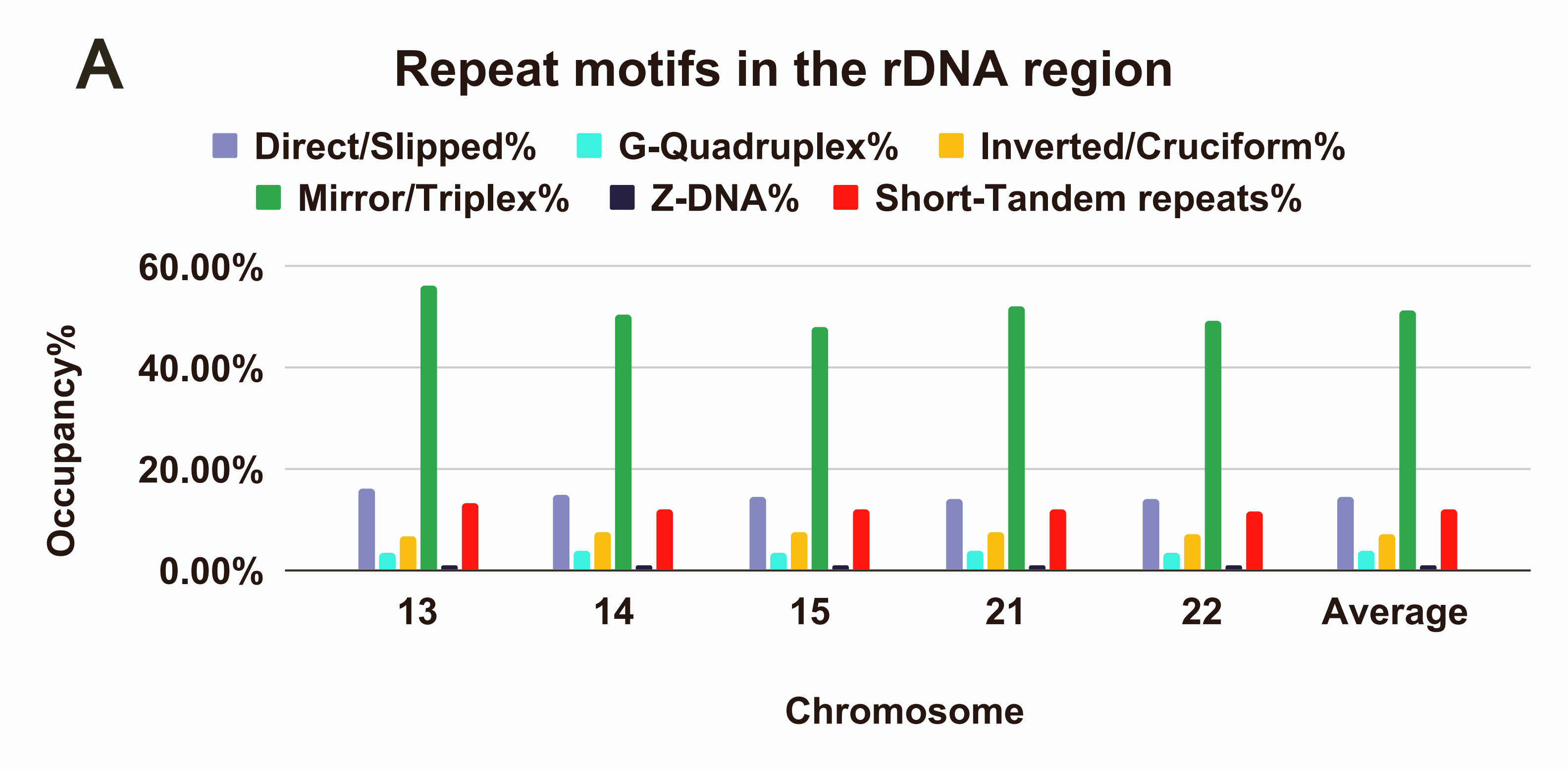
A
Repeat motifs in the rDNA region
Direct/Slipped%
G-Quadruplex%
Inverted/Cruciform%
Mirror/Triplex%
Z-DNA%
Short-Tandem repeats%
60.00%
40.00%
20.00%
0.00%
Occupancy%
13
14
15
21
22
Average
Chromosome

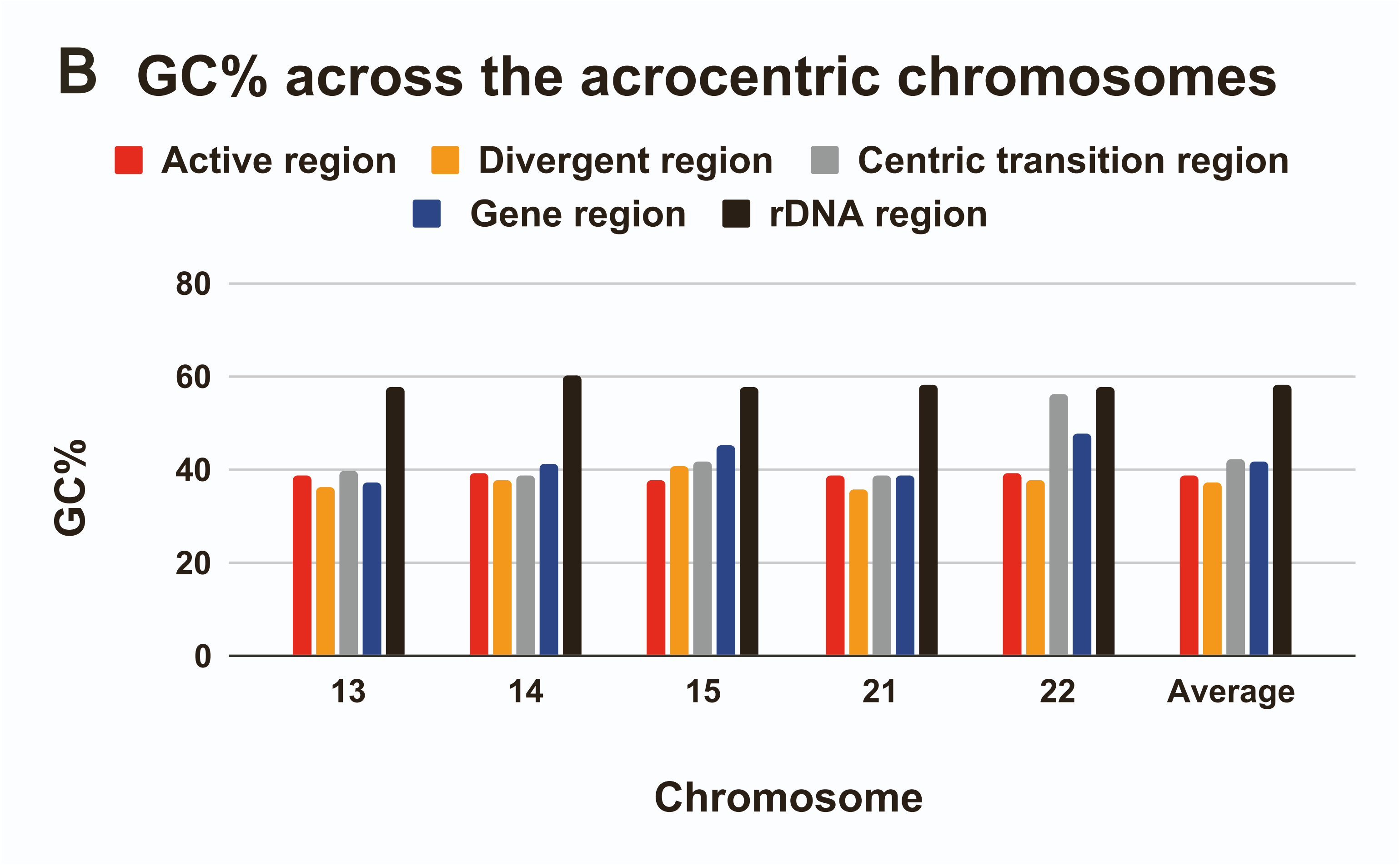
B
GC% across the acrocentric chromosomes
Active region
Divergent region
Centric transition region
Gene region
rDNA region
80
60
40
20
0
GC%
13
14
15
21
22
Average
Chromosome

FIGURE S4

Figure S4. Acrocentric chromosomes comparison. (A) Occupancy of repeat motifs, namely, Direct repeats, G4 motifs, Inverted repeats, Mirror repeats, Z-DNA motifs and Short tandem repeats in the rDNA region. (B) Comparison of GC-content in the rDNA region against the peri/centromeric regions as well as the gene region.

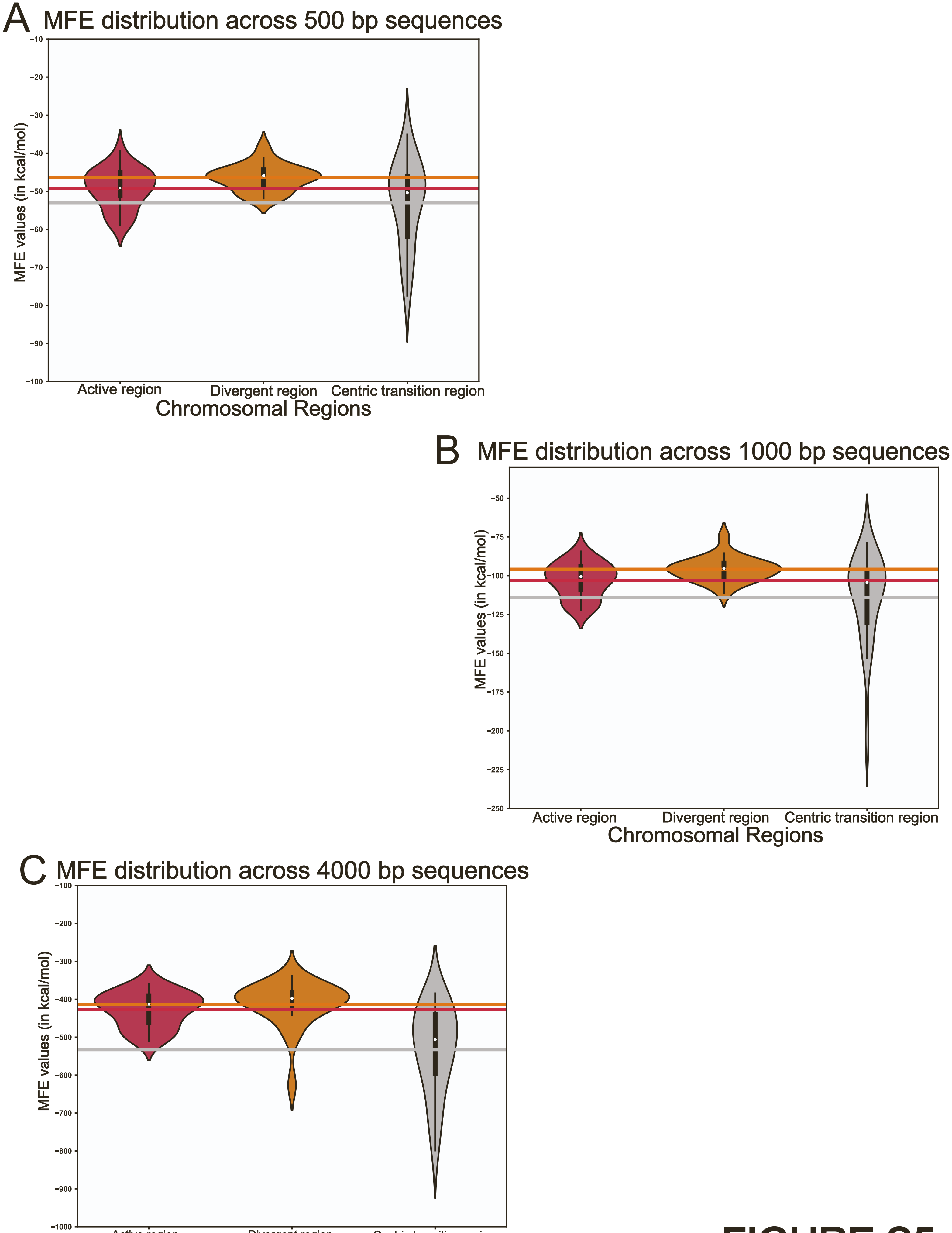
A
MFE distribution across 500 bp sequences
MFE values (in kcal/mol)
−10
−20
−30
−40
−50
−60
−70
−80
−90
−100
Active region
Divergent region
Centric transition region
Chromosomal Regions
B
MFE distribution across 1000 bp sequences
MFE values (in kcal/mol)
−50
−75
−100
−125
−150
−175
−200
−225
−250
Active region
Divergent region
Centric transition region
Chromosomal Regions
C
MFE distribution across 4000 bp sequences
MFE values (in kcal/mol)
−100
−200
−300
−400
−500
−600
−700
−800
−900
−1000
Active region
Divergent region
Centric transition region
Chromosomal Regions

FIGURE S5

Figure S5. MFE value distribution across centromeric sequences of varying lengths. (A) A violin plot displaying the spread of minimum free energy values in the 500bp sequences in the active region, the divergent region and the centric transition region. The horizontal lines correspond to the mean MFE value in each region. (B) A violin plot displaying the spread of minimum free energy values in the 1000bp sequences in the active region, the divergent region and the centric transition region. The horizontal lines correspond to the mean MFE value in each region. (C) A violin plot displaying the spread of minimum free energy values in the 4000bp sequences in the active region, the divergent region and the centric transition region. The horizontal lines correspond to the mean MFE value in each region.

A RPE-1 HAPLOTYPE 1: |MFE| VS PROBABILITY OF MISSEGREGATION

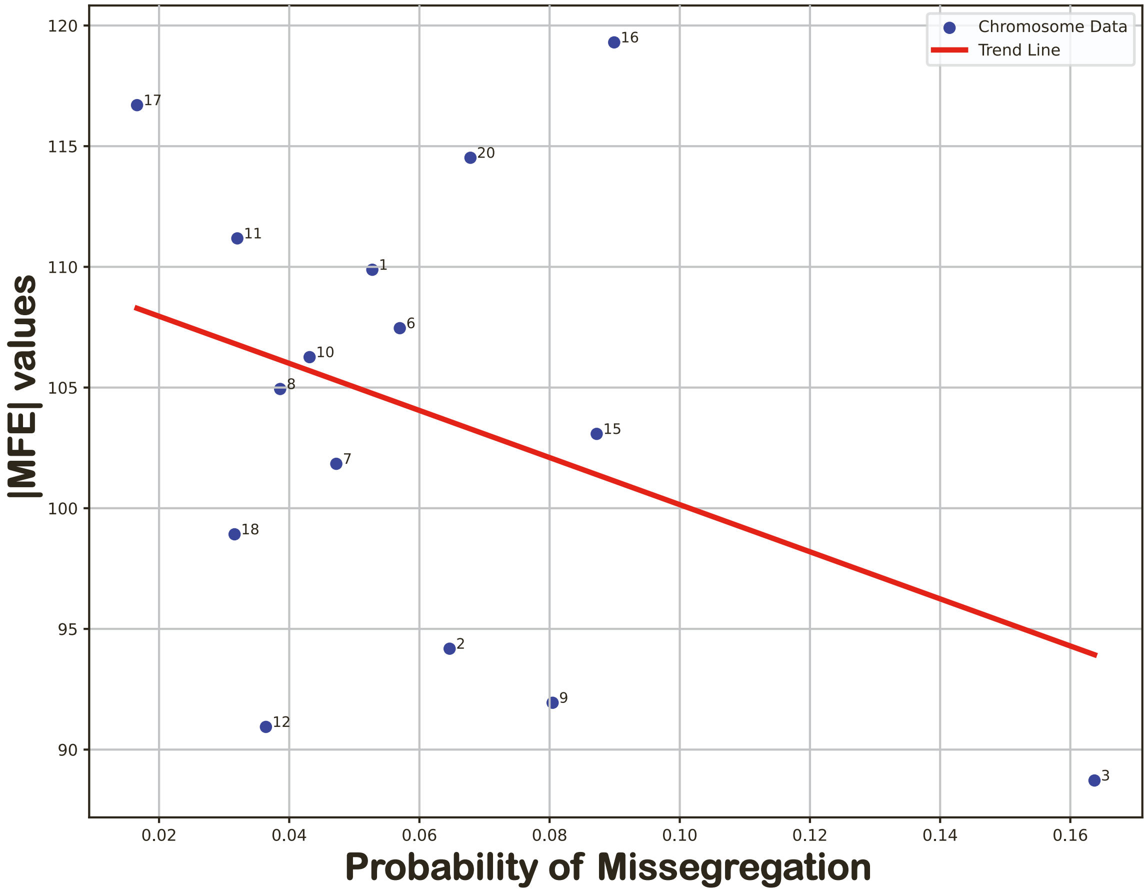

B RPE-1 HAPLOTYPE 2: |MFE| VS PROBABILITY OF MISSEGREGATION

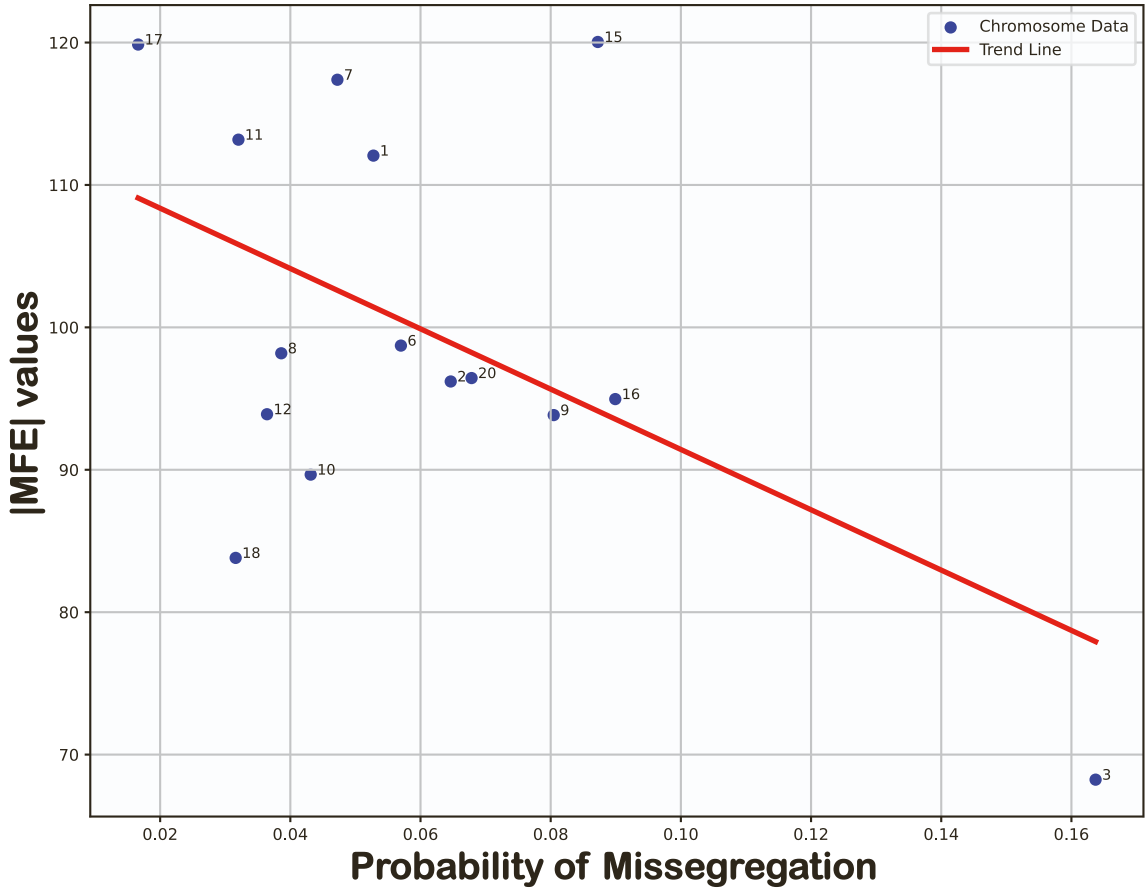

FIGURE S6

Figure S6. Correlation between the |MFE| values and the probability of missegregation. (A) Scatterplot showing the probability of missegregation on the x-axis and the |MFE| values of the secondary structures predicted from sequences extracted from the active region of RPE1 haplotype 1 on the y-axis. A correlation of -0.36 was found between the values on the x-axis and the y-axis. (B) Scatterplot showing the probability of missegregation on the x-axis and the |MFE| values of the secondary structures predicted from sequences extracted from the active region of RPE1 haplotype 2 on the y-axis. A correlation of -0.52 was found between the values on the x-axis and the y-axis. The missegregation probability values were obtained from [1].

Tables summary

Table S1 – Sequences and derived data evaluated in the study for linear DNA of 4000 bp.

Tabs:

1. Sequence Data: DNA sequences extracted and utilized in this analysis along with their genomic coordinates and their region information.
2. Thermodynamic Data: thermodynamic stability data, namely, the Minimum free energy values, the free energy of the thermodynamic ensemble values and the ensemble diversity values of the secondary structures predicted from the DNA sequences present in Sequence Data spreadsheet. This data was obtained using the tool RNAfold [2].
3. Ensemble Diversity Pivot Table: Ensemble Diversity data pivot table displaying the average ensemble diversity values of DNA sequences from all the chromosomes considered across the regions under study. This data was obtained using the tool RNAfold [2].
4. FETE Pivot Table: Free energy of the Thermodynamic Ensemble data pivot table displaying the average free energy of the thermodynamic ensemble values of DNA sequences from all the chromosomes considered across the regions under study. This data was obtained using the tool RNAfold [2].
5. Probability_of_missegregation: Spreadsheet containing the information regarding the probability of missegregation across multiple chromosomes. This data was obtained from [1].
6. MFE Pivot Table: Minimum free energy data pivot table displaying the average minimum free energy values of DNA sequences from all the chromosomes considered across the regions under study. This data was obtained using the tool RNAfold [2].

Table S2 – Non-B DNA output data evaluated in the study.

Tabs:

1. Active region: information on the various non-B DNA motifs detected in the DNA sequences in the active HOR region. This information was obtained using the tool Non-B DNA search tool (nBDST) [3]
2. Divergent region: information on the various non-B DNA motifs detected in the DNA sequences in the divergent HOR region. This information was obtained using the tool Non-B DNA search tool (nBDST) [3]
3. Centric Transition region: information on the various non-B DNA motifs detected in the DNA sequences in the centric transition region. This information was obtained using the tool Non-B DNA search tool (nBDST) [3]
4. rDNA region: information on the various non-B DNA motifs detected in the DNA sequences in the rDNA region. This information was obtained using the tool Non-B DNA search tool (nBDST) [3]

Table S3 – Dyad density and symmetry evaluated in the study.

Tabs:

1. Dyad Symmetries: density of dyad symmetries found in the peri/centromeric regions, namely, the active HOR, the divergent HOR and the centric transition region. This data was obtained using the tool Palindrome by EMBOSS [4].
2. Dyad Symmetries Pivot Table: pivot table displaying the average dyad density in the peri/centromeric regions, namely, the active HOR, the divergent HOR and the centric transition region in the chromosomes considered for this study. This data was obtained using the tool Palindrome by EMBOSS [4].

Table S4 – Sequences and GC content evaluated in the study.

Tabs:

1. GC%: the genomic coordinates of the DNA sequences extracted along with the region information, sequence information and the GC%.
2. GC% Pivot Table: pivot table containing the average GC% values observed in the DNA sequences extracted from various chromosomes across the various genomic regions considered in our study.

<u>Table S5 – Sequences and data derived and evaluated in the study for linear DNA of 500 and 1000 bp.</u>

Tabs:

1. CHM13 500bp sequences: the genomic coordinates of the 500bp sequences extracted along with their chromosome, region and sequence information. This spreadsheet also contains thermodynamic stability data, namely, the minimum free energy (MFE) values, free energy of the thermodynamic ensemble (FETE) values and ensemble diversity (ED) values pertaining to the secondary structures predicted to form by these DNA sequences. The thermodynamic stability data was obtained using the tool RNAFold [2].
2. CHM13 500bp sequences MFE Pivot: pivot table containing the average MFE values of the 500bp sequences extracted from the peri/centromeric regions, namely, the active HOR region, the divergent HOR region and the centric transition region. The thermodynamic stability data was obtained using the tool RNAFold [2].
3. CHM13 1000bp sequences: the genomic coordinates of the 1000bp sequences extracted along with their chromosome, region and sequence information. This spreadsheet also contains thermodynamic stability data, namely, the minimum free energy (MFE) values, free energy of the thermodynamic ensemble (FETE) values and ensemble diversity (ED) values pertaining to the secondary structures predicted to form by these DNA sequences. The thermodynamic stability data was obtained using the tool RNAFold [2].
4. CHM13 1000bp sequences MFE Pivot: pivot table containing the average MFE values of the 500bp sequences extracted from the peri/centromeric regions, namely, the active HOR region, the divergent HOR region and the centric transition region. The thermodynamic stability data was obtained using the tool RNAFold [2].

Table S5 – RPE-1 Chromosome missegregation analyses data.

Tabs:

1. RPE1Hap1_active_region_MFE: This spreadsheet contains the genomic coordinates of the 1000bp long DNA sequences extracted from the active HOR region of the centromere in the Haplotype 1 of the RPE1 genome. This spreadsheet also holds the sequence data and the thermodynamic stability information, namely, the minimum free energy (MFE), free energy of the thermodynamic ensemble (FETE) and ensemble diversity (ED) of the secondary structures these DNA sequences are predicted to form. The thermodynamic stability data was obtained using the tool RNAFold [2].
2. RPE1Hap1 MFE Pivot table: This spreadsheet is a pivot table displaying the average MFE, FETE and ED values of the secondary structures predicted to form by the DNA sequences extracted from the active HOR region of chromosomes considered. The thermodynamic stability data was obtained using the tool RNAFold [2].
3. RPE1Hap1_MFE vs Missegregation: This spreadsheet displays the average MFE values of secondary structures predicted to form from the DNA sequences of the active HOR region in haplotype 1 of the RPE-1 genome next the probability of missegregation information. The data is sorted in ascending order in regard with the probability of missegregation. The missegregation probability information was obtained from [1].
4. RPE1Hap2_active_region_MFE: This spreadsheet contains the genomic coordinates of the 1000bp long DNA sequences extracted from the active HOR region of the centromere in the Haplotype 2 of the RPE1 genome. This spreadsheet also holds the sequence data and the thermodynamic stability information, namely, the minimum free energy (MFE), free energy of the thermodynamic ensemble (FETE) and ensemble diversity (ED) of the secondary structures these DNA sequences are predicted to form. The thermodynamic stability data was obtained using the tool RNAFold [2].
5. RPE1Hap2 MFE Pivot table: This spreadsheet is a pivot table displaying the average MFE, FETE and ED values of the secondary structures predicted to form by the DNA sequences extracted from the active HOR region of chromosomes considered in haplotype 2 of the RPE-1 genome. The thermodynamic stability data was obtained using the tool RNAFold [2].
6. RPE1Hap2_MFE vs Missegregation: This spreadsheet displays the average MFE values of secondary structures predicted to form from the DNA sequences of the active HOR region in haplotype 2 of the RPE-1 genome next the probability of missegregation information. The data is sorted in ascending order in regard with the probability of missegregation. The missegregation probability information was obtained from [1].

References

1. Dumont M, Gamba R, Gestraud P, Klaasen S, Worrall JT, De Vries SG, et al. Human chromosome-specific aneuploidy is influenced by DNA-dependent centromeric features. EMBO J 2020;39:e102924. https://doi.org/10.15252/embj.2019102924.
2. Lorenz R, Bernhart SH, Höner Zu Siederdissen C, Tafer H, Flamm C, Stadler PF, et al. ViennaRNA Package 2.0. Algorithms Mol Biol 2011;6:26. https://doi.org/10.1186/1748-7188-6-26.
3. Cer R, Bruce K, Donohue D, Temiz N, Mudunuri U, Yi M, et al. Searching for non-B DNA-forming motifs using nBMST (non-B DNA Motif Search Tool). Curr Protoc Hum Genet 2012;CHAPTER:Unit-18.722. https://doi.org/10.1002/0471142905.hg1807s73.
4. [36] Rice P, Longden I, Bleasby A. EMBOSS: the European Molecular Biology Open Software Suite. Trends Genet 2000;16:276–7. https://doi.org/10.1016/s0168-9525(00)02024-2.
