# Supplementary material for: Comparative analysis of predicted DNA secondary structures infers complex human centromere topology
Source: Am J Hum Genet. 2024 Nov 18;111(12):2707–19. doi: 10.1016/j.ajhg.2024.10.016 (PMC11639080; doi:10.1016/j.ajhg.2024.10.016)
Supplement: Document S2. Article plus supplemental information [file mmc8.pdf]

Comparative analysis of predicted DNA secondary structures infers complex human centromere topology

Graphical abstract

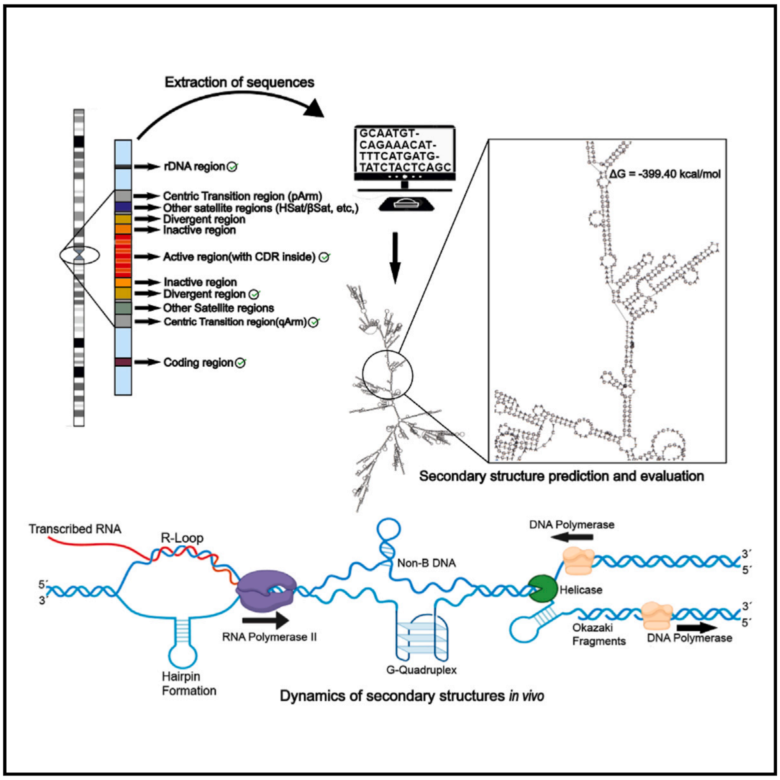

Authors

Sai Swaroop Chittoor, Simona Giunta

Correspondence

simona.giunta@uniroma1.it

DNA-based transactions can unwind the double helix, promoting the formation of secondary structures through intra-strand interactions and non-canonical folding. Our study predicts that human centromere sequences harbor more of these complex and unstable DNA structures than other genomic regions, potentially driving chromosome segregation errors and genomic instability.

Chittoor & Giunta, 2024, The American Journal of Human Genetics *111*, 2707–2719
December 5, 2024 © 2024 The Authors. Published by Elsevier Inc. on behalf of American Society of Human Genetics.
https://doi.org/10.1016/j.ajhg.2024.10.016

Comparative analysis of predicted DNA secondary structures infers complex human centromere topology

Sai Swaroop Chittoor[1] and Simona Giunta[1,*]

Summary

Secondary structures are non-canonical arrangements of nucleic acids due to intra-strand interactions, including base pairing, stacking, or other higher-order features that deviate from the standard double-helical conformation. While these structures are extensively studied in RNA, they can also form when DNA becomes single stranded, creating topological roadblocks that can impact essential DNA-based processes such as replication, transcription, and repair, ultimately affecting genome stability. The availability of a complete linear sequence of human genomes, including repetitive loci, enables the prediction of DNA secondary structures comparing across various regions. Here, we evaluate the intrinsic properties of linear single-stranded DNA sequences derived from sampling specialized human loci such as centromeres, pericentromeres, ribosomal DNA (rDNA), and coding regions from the CHM13 genome. Our comparative analysis of predicted secondary structures across human chromosomes revealed the heightened presence, complexity, and instability of secondary structures within the centromere, which gradually decreased toward the pericentromere onto chromosomes' arms, on average lowest in coding regions. Notably, centromeric repeats exhibited the highest level of topological complexity within both the active and divergent domains, even when compared to other repetitive tandem satellites, such as rDNA in acrocentric chromosomes. Our findings provide evidence of the intrinsic self-hybridizing properties of centromere repeats, which are capable of generating complex topological structures that may functionally correlate with chromosome missegregation, especially when centromeric chromatin is disrupted. Processes such as long non-coding RNA transcription, recombination, and other mechanisms that dechromatinize and unwind stretches of linear DNA in these regions create *in vivo* opportunities for the DNA acrobatics hereby predicted.

Introduction

The centromere is an essential genomic region whose position within the primary constriction has been used to classify chromosomes (Figures S1A and S1B). In addition to representing a visually distinct part of eukaryotic chromosomes, centromeres facilitate sister chromatids' cohesion as well as act as the basal layer for the formation of the proteinaceous structure called the kinetochore, which physically associates the centromere to the spindle fibers during the process of chromosome division.[1,2]

Due to their long length and highly repetitive nature made of near-identical repeats called higher-order repeat (HOR) arrays, obtaining the complete linear sequence information of human centromeres has been a long-standing technical challenge.[3–5] The recent CHM13 assembly of the human genome utilized third-generation sequencing technologies like PacBio High Fidelity (HiFi) and Oxford Nanopore Ultralong (ONT) to achieve long DNA reads that enabled a comprehensive map of a gapless human genome of a pseudo-haploid cell line derived from an inviable molar pregnancy.[6] Previously missing regions of the genome were assembled, the remaining gaps spanning areas containing long repetitive sequences like the centromeric region were filled in, and the rDNA arrays were modeled. Ultimately, the availability of these linear sequences has unlocked an opportunity to study the base pair composition as well as the structure of DNA throughout the human genome.

A large portion of the genome comprises repetitive sequences,[7] of which the centromere and pericentromere make up approximately 6%–8% (~189 Mb in CHM13).[6,8] Centromeres are composed of long arrays of repeating sequences called satellite DNA, particularly alpha-satellites, which have a basic repeated unit an ~170 bp monomer. These alpha-satellites arranged tandemly in HOR units make up the core of the centromere, spanning up to ~7 Mb as one of the longest centromeres assembled.[9] Centromeres rarely contain protein-coding genes but are actively transcribed into long non-coding centromeric RNAs (cenlncRNAs).[10] The region where HOR arrays share a high level of sequence identity is referred to as active or live centromere with high-density enrichment of the centromere protein A (CENP-A).[8,9] CENP-A is a histone H3 variant that epigenetically marks the binding site of the kinetochore and, thus, the epigenetic position of the centromere on the chromosome.[11] Correct kinetochore attachments are essential in preventing mitotic dysfunction and chromosome missegregation.

Flanking the active centromere, the divergent region transitions into the pericentromere with progressively more divergent HORs containing non-uniform repeat units. On either side flanking the centromere progressing into the chromosome's arms, we can find other types of

[1]Laboratory of Genome Evolution, Department of Biology and Biotechnology Charles Darwin, Sapienza University of Rome, 00185 Rome, Italy
*Correspondence: simona.giunta@uniroma1.it
https://doi.org/10.1016/j.ajhg.2024.10.016.
© 2024 The Authors. Published by Elsevier Inc. on behalf of American Society of Human Genetics.
This is an open access article under the CC BY license (http://creativecommons.org/licenses/by/4.0/).

satellite DNA depending on the chromosome, such as (1) human satellites HSat1A and HSat1B, which constitute the most AT-rich regions in the chromosome, (2) HSat2 and HSat3, which derive from a (CATC)n repeat sequence, and (3) beta-satellites and gamma-satellites, which are GC-rich stretches containing dense CpG methylation.[8] The various satellite regions in the pericentromere are usually followed by a stretch of non-satellite sequences termed the centric transition region. The centric transition also flanks the pericentromere, separating it from the gene-coding unique p and q arms of the chromosomes. In CHM13, the centric transition region contains segmental duplications while occasionally housing lncRNAs as well as protein-coding genes (e.g., *ADAP2* [MIM: 608635] in the q arm of chromosome 17).[8]

DNA in its native state exists as a double-stranded, right-handed helix, as described by Crick and Watson[12] based on the experimental data of Rosalind Franklin.[13] This DNA canonical conformation is termed B-DNA and is considered one of the most stable forms of DNA and the most common biologically occurring. While strong hybridization of the two complementary strands and chromatinization largely protect DNA from aberrant self-annealing, when DNA is single stranded, it opens opportunities for intrastrand base pairing due to complementarity, leading to the formation of unimolecular folded structures in a fashion similar to that observed for RNA.[14] Hence why the prediction of secondary structures has historically been done on RNA, with the initial predictions of RNA secondary structures in the early 1970s done using simple energy models.[15] While DNA structures are thermodynamically unfavorable in the linear form, mounting evidence points to cases of secondary-structure formation during biological processes like replication, transcription, DNA repair, or other nuclear processes that require opening of the DNA concomitant with dechromatinization—where the DNA is unwrapped from the nucleosomes, base complementarity within the antiparallel strand is broken, and complementarity within the same strand can favor the dynamic formation of secondary structures.[16] Such secondary structures can also be formed due to the binding of specific proteins to the DNA molecule or due to changes in temperature, pH, and salt concentration. Once formed, single-stranded secondary structures may support the formation of alternative DNA conformations collectively referred to as non-B DNA.[17] Non-B DNA can arise due to various factors such as the presence of specific DNA motifs, changes in the environmental conditions, or the action of certain proteins on the DNA. Non-B DNA structure formation has been widely reported in repetitive DNA sequences like G-quadruplexes, formed by the repetition of guanine nucleotides in immunoglobulin class-switch recombination (CSR) regions and telomeres.

Non-B DNA presence and enrichment at the centromere have long been observed, with the formation of hairpins and cruciform structures mainly attributed to dyad symmetries.[18–20] It has been speculated to be one of the defining factors of the centromere since both the centromeres and neocentromeres are enriched for inverted repeats, which possess the potential to form non-B DNA structures,[18] and are notoriously difficult to sequence through.[21,22] Consistent with the presence of non-B-form DNA structures at functional centromeres, DNA hairpins, triplexes, and R-loops have been observed in alpha-satellite DNA *in vitro* and/or *in vivo*.[23–25] Notably, centromeres have been suggested to be akin to common fragile sites of the human genome,[23,26] where late replication,[26,27] presence of tandem repeats,[28] and active mitotic recombination[29] converge to promote DNA instability.[26] Indeed, upon CENP-A removal and chromatin perturbation, R-loop formation at centromeres leads to DNA breaks, impacting the stability of the DNA in the region and leading to chromosome arm aneuploidy.[23]

In this work, we sought empirical evidence of centromeric DNA forming secondary structures by sampling DNA sequences from the human genome across chromosomes. We opted to utilize the tool RNAfold from the ViennaRNA package for our analysis due to its proven track record in terms of accuracy and runtime optimization for DNA secondary-structure prediction and minimum free energy (MFE) calculation compared to other available tools.[30] RNAfold uses a loop-based energy model (Figure 1A) and Zuker's algorithm,[31] integrating experimentally evaluated and compiled nearest-neighbor parameters.[32,33] Our data revealed a high inherent intricacy of the DNA sequence in the core centromere as inferred from the complex nature of the secondary structures predicted, whose stabilities progressively increase toward the chromosome arm. This is mirrored by the sequence composition of the DNA as well as the repeat organization of centromeric DNA. We also compared the peri/centromeric DNA against rDNA, another repetitive region in the acrocentric chromosomes, as well as against functional genes across chromosomes, uncovering relative differences among their secondary structures. The complexity in centromere secondary structures correlates with the rate of missegregation of individual chromosomes, in both CHM13 and RPE-1 genome assemblies,[6,9] hinting at a functional impact of complex DNA topologies. Altogether, our study offers an overview of the secondary-structure-forming capabilities of the human genome based on linear DNA sequences starting from the core centromere and along chromosome arms.

Material and methods

Sequence information

All the sequence information used in this analysis was obtained from the CHM13 human genome assembly[6] unless specified otherwise. From the active HOR region, divergent HOR region, centric transition region, and rDNA region, we isolated five DNA sequences in all the human chromosomes except chromosome 4 and chromosome X, which do not possess a sufficiently large divergent region within the centromere in the CHM13 genome.

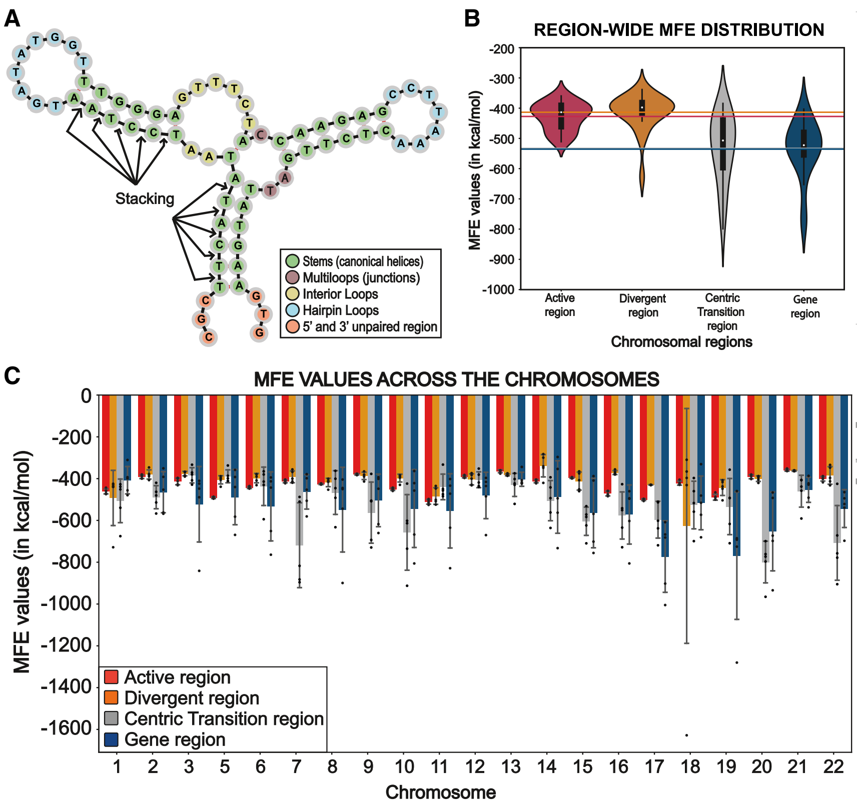

Figure 1. An overview of MFE value distribution from human chromosomes

(A) Diagram of the parameters assessed in this study, with a DNA secondary structure broken down into loops containing unpaired bases like multiloops (point of connection between three or more helical stems), hairpin loops (unpaired region of a hairpin alongside a double-stranded stem), or interior loops (regions that link exactly two stems)[34] and stacking complementary base pairs. The ΔG value of a DNA secondary structure is evaluated as the sum of the contributing free energies of stabilizing factors (complementary base pairs and corresponding base pair stackings) and destabilizing factors (loops, bulges, unpaired bases) contained in the secondary structure. This figure was created using forna.[35]

(B) Violin plot visualization of the spread of the average MFE values across regions of the chromosome as indicated. The horizontal lines represent the mean for each region.

(C) Bar plot displaying the spread of the average MFE values of the predicted secondary structures from the selected regions. Bars contain 5 dots that correspond to the original MFE data values from the five different regions sampled. The x axis represents the chromosomes, and the y axis represents their minimum free energy values in kcal/mol. The error bars represent the standard deviation. Chromosome 4 was omitted because it lacks a divergent region, and chromosome X was omitted because it possesses a divergent region <4,000 bp.

To unbiasedly select the DNA sequences to be analyzed, we applied the method of stochastic sampling and randomly selected five sequence fragments from the aforementioned regions (https://github.com/GiuntaLab/DNA-secondary-structures-analysis). We performed the analysis using DNA of 4,000 bp in length based on the assumption that lncRNA generated through the transcription of human DNA repeats can extend for more than 1 kb, as recently shown.[36] To assess whether the length of the DNA may artifactually impact our secondary-structure predictions and the derived values, we extended our sampling to other sequences within the chosen regions that span 500 bp and 1 kb in length. As expected, the MFE values change according to the number of bases in the sequence, yet the trend for secondary-structure complexity was the same whether we used 4,000, 1,000, or 500 nucleotide sequences (Figures S5A–S5C). These data confirm that the MFE values compared across different regions in the peri/centromere of the human genome are unaffected by the length of DNA for the three sizes we assayed, and the trends we have identified represent *bona fide* indicators to predict secondary structures based on topological organization of nucleotide sequences. For the coding regions, we selected five random functional protein-coding genes in the q arm of the chromosome and extracted DNA sequences of length 4,000 bp from every gene selected.

The selection of protein-coding genes as well as their sequences was done in a random manner to assess how the selected sequences would fare in terms of secondary structures and MFE values when juxtaposed with the DNA sequences selected from the peri/centromeric regions and other repetitive DNA. Due to this random assignment, they do not truly represent all the protein-coding genes and may be subject to selection bias.

Secondary structure prediction and delta G value calculation

The DNA secondary-structure prediction, as well as the calculation of MFE values, was done using the tool RNAfold from the ViennaRNA package (v.2.5.1; https://github.com/ViennaRNA/ViennaRNA).[30] Predictions were done using the following parameters:

```
RNAfold -d2 -g –noLP -P dna_mathews2004.par –noconv
```

Predictions were performed on samples of 500, 1,000, and 4,000 bp sequences extracted from 5 different regions within the active HOR, divergent HOR, centric transition region, randomly selected sequences from the protein-coding region in the q arm of the chromosome, and the rDNA.

Non-B DNA motif prediction

The prediction of occurrence of inverted repeats as well as various other non-B DNA motifs in the regions considered was done using the Non-B DNA Motif Search Tool (nBMST; https://nonb-abcc.ncifcrf.gov/apps/nBMST).[37] The following command was used in this analysis to generate the output:

```
gfa -seq input.fasta -out output
```

The results were then normalized by dividing the sum of lengths of every non-B DNA motif by the total size of the DNA regions considered.

This tool can accurately generate the predictions of seven different non-B DNA-forming motifs: A-phased DNA repeats (bent DNA), direct repeats (slipped structures), mirror repeats (triplex DNA), inverted repeats (cruciform structures), alternating purine-pyrimidine tracts (Z-DNA), G4 motifs (G-quadruplexes), and short tandem repeats.

Dyad density evaluation

For the identification of small dyad symmetries across the entire peri/centromeric loci, we used EMBOSS Palindrome (https://www.bioinformatics.nl/cgi-bin/emboss/palindrome)[38] with the following parameters:

```
palindrome -sequence input.fasta -minpallen 5 -maxpallen 100
-gaplimit 20 -nummismatches 0 -overlap
```

Following this, we computed the dyad density by summing the lengths of all palindromic regions identified and then dividing this sum by the length of the input DNA sequence.

Results

DNA secondary-structure prediction and MFE calculation

Unique higher-order chromatin and DNA structures may be specific to different human loci and repetitive regions.[39] To test the self-hybridizing properties of DNA regions *in silico*, we utilized specific linear DNA sequences from the CHM13 human genome assembly.[6] We assessed the thermodynamic stability of the Gibbs free energy surrounding the secondary structures predicted to form based on the DNA sequences belonging to various regions. Gibbs free energy is a concept in thermodynamics that combines both the enthalpy (heat energy) and entropy (disorder) contributions of a system.[40] During DNA folding, a single-stranded DNA molecule can undergo a series of structural conformations, each associated with specific changes in Gibbs free energy (ΔG). The molecule will ultimately settle on a secondary structure that possesses the lowest ΔG value since the folding of DNA is driven by the principle of free energy minimization. This structure is termed the MFE structure, and its free energy is the lowest among all the possible structures in the ensemble. The lower the free energy value, the higher the stability of the folded secondary structure of the DNA.

The main energy contributors are base pair stacking due to complementary base pairing, stacking interactions, sequence composition, the destabilizing entropic effects of unpaired loops, and the presence of structural motifs (Figure 1A).[30] Since ionic conditions, temperature, and pH influence the energy contributions of the elements in the system, we performed the analysis using DNA sequences of equal length simulated at a standard temperature value of 37°C (310 K) and a fixed salt concentration of 1.021 M NaCl.

We jointly assessed the secondary structures along with their corresponding MFE values comparing 4 regions from the centromere and pericentromere and unique coding sequences in human autosomes. As expected, in the absence of repetitive DNA, the MFE value was found to be the lowest in the gene regions (Table S1; Figures 1B and 1C), due to a lack of complementary neighboring sequences that provide an opportunity for self-hybridization should DNA become single stranded. The MFE increased as we sampled toward the centric transition region in the pericentromere and peaked at the divergent and active HOR region of the centromere (Figure 1B). The MFE values of the divergent HOR stand out as the highest among all the regions considered, indicating the very low stability of the predicted DNA secondary structures with an MFE value average of −413.43 kcal/mol, followed by −427.47 kcal/mol for the active HOR, −533.06 kcal/mol for the centric transition region of the pericentromere and −535.47 kcal/mol for coding loci, indicating a notable but non-significant difference in their structural stability (Figure 1B). As observed in the violin plot (Figure 1B), the MFE values of the secondary structures predicted in the active and divergent HOR DNA are less variable and cluster together, indicating a high level of complexity in the secondary structures across different chromosomes.

Concurrently, we noticed a higher frequency of various structural organizations to be more prominent in the centromere, including short hairpins with large stem loops, complex branching hairpins, large bulges, and loops in the secondary structures with a high MFE value—for both active and inactive HOR sequences. A drastic drop in the complexity of structural motifs and an increase in linearity were observed for the pericentromeric region and within the gene regions. This further substantiated our findings on how structural motifs may directly fuel the complexity and instability of a secondary structure (Figure 2).

We next analyzed whether a chromosome or chromosome subgroup was primarily responsible for the average MFE of that region, especially for coding and pericentromere DNA, where the spread of the MFE has high variation (Figure 1C). While the overall trend is evident in the average values across all the chromosomes, there are examples in which individual chromosomes deviate from these expectations. For example, despite having the highest average MFE across all the chromosomes, the secondary structure with the lowest MFE value of −1628.1 kcal/mol belongs to the DNA from the divergent HOR region in chromosome 18, making it the most stable sequence sampled (Figure S2). This is to be expected because we randomly sampled 5 different 4,000 nucleotide sequences within each region for each chromosome starting from the beginning of the BED file coordinates annotated for that region. Altogether, our evidence shows the high complexity of predicted DNA secondary structures in the human centromere that progressively decreases into flanking pericentromeres and further along the chromosome arm in our sampled coding sequences.

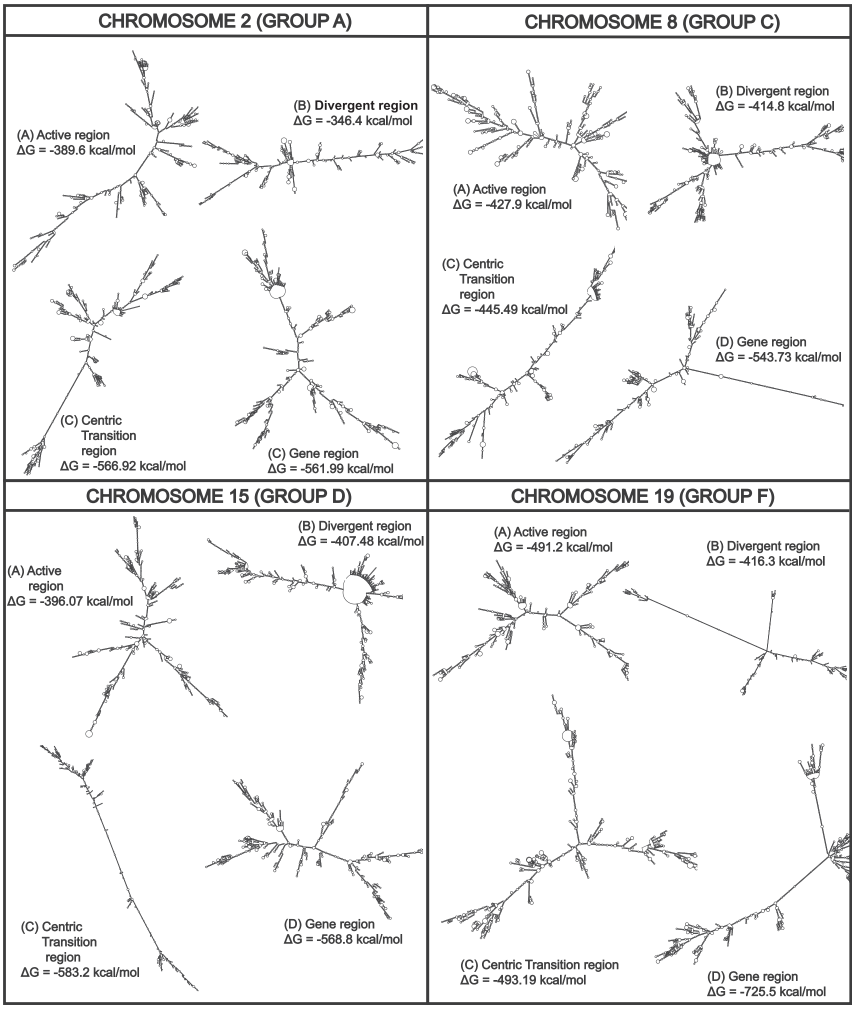

Figure 2. Examples of predicted secondary structures and their stabilities
Examples of predicted secondary structures, as well as their stabilities, calculated from the DNA sequences for chromosomes from four different groups, namely chromosome 2 from group A, representing the metacentric chromosomes; chromosome 8 from group C, representing the submetacentric chromosomes; chromosome 15 from group D, representing the acrocentric chromosomes; and finally, chromosome 19 from group F, representing the short metacentric chromosomes. Arrows indicate examples of branching stems.

Centromere arrays show the highest diversity of DNA secondary-structure ensembles

Next, we evaluated two additional parameters associated with DNA secondary structures: the free energy of the thermodynamic ensemble and ensemble diversity. The free energy of the thermodynamic ensemble is defined as the average free energy of all possible secondary structures and provides a measure of the overall stability landscape of the DNA sequence. Ensemble diversity is defined as the average base pair distance between all structures in the thermodynamic ensemble. A low ensemble diversity indicates a few similar conformations, while higher ensemble diversity suggests multiple diverse conformations or a lack of a defined structure.[41,42] We observed that the free energy of the thermodynamic ensembles across the various regions follows the same trend as the MFE values of the predicted secondary structures, with the thermodynamic ensemble values in active and divergent centromeres clustering together, whereas the data values for the centric transition regions and gene region were more variable (Figures 3A and 3B; Table S1). Similarly, we noticed that the ensemble diversity data also validated our findings regarding complexity in secondary structures, in line with the MFE values, with the active region showing the highest ensemble diversity, signifying a lack of stable structures or possibly the presence of multiple unstable secondary structures. This is somewhat unexpected, as near-identical homogeneous centromere repeats should, in theory, be more prone to form the same secondary structures many times, while our data suggest that the MFE value (Figure 1B) is likely due to many different secondary structures without a single consensus, as their repeated sequence organization would suggest, or multiple options for different secondary structures formed by the same sequence. Notably, the DNA belonging to the centric transition region has the lowest ensemble diversity value, indicating the presence of stable secondary structures in the thermodynamic ensemble (Figures 3C and 3D; Table S1) and, accordingly, the low MFE (Figure 1B).

Short dyad symmetries have been observed in the centromeric DNA of various species.[18–20] These sequences directly promote the formation of inverted repeats and unconventional secondary structures since they are composed of a DNA sequence followed by its reverse complement, separated by a spacer. We found a consistent inverted repeat occupancy rate in the peri/centromeric regions (~4.25% occupancy rate) (Table S2; Figures S3A–S3C). Our data are in line with the latest studies on Y chromosome assembly,[22,43] where inverted repeats were identified as potentially forming cruciforms and playing a functional role in defining human Y centromeres.[22] Beyond chromosome Y, our analysis revealed the presence of inverted repeats across centromeres and pericentromeres of all chromosomes analyzed. This is particularly interesting in light of inverted repeat sequences (IRs) colocalizing with breakage hotspots and contributing to deletions, amplifications, and translocations, with ensuing chromosomal instability.[44] This is consistent with our

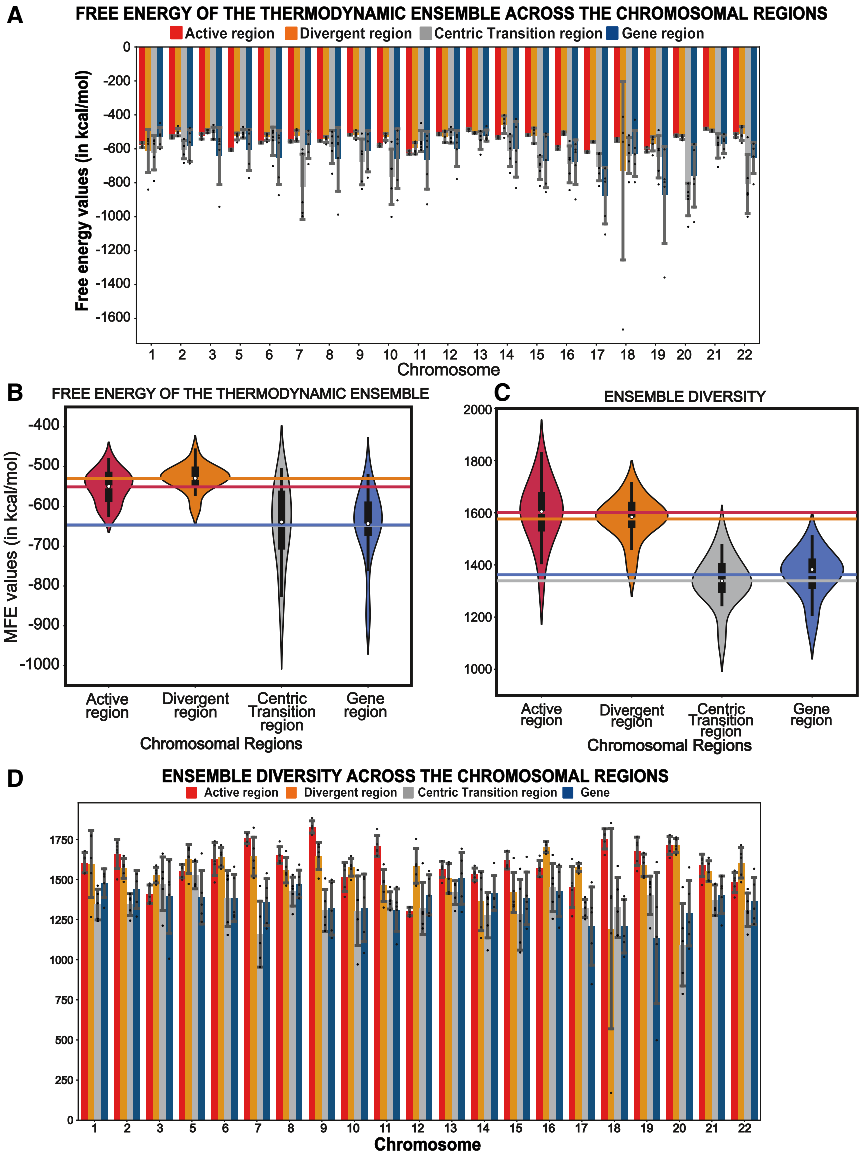

Figure 3. Evaluation of additional secondary-structure parameters

(A) Bar plot displaying the spread of free energy of the thermodynamic ensemble average values of the predicted secondary structures from the selected regions. Each bar contains 5 dots that correspond to the original free energy of the thermodynamic ensemble values. The x axis represents the chromosomes, and the y axis represents their free energy values in kcal/mol. The error bars represent the standard deviation.
(B) A violin plot showcasing the spread of the ensemble free energy average across the four regions. The horizontal lines correspond to the mean for each region.
(C) A violin plot displaying the distribution of average ensemble diversities. The horizontal lines correspond to the mean for each region.
(D) Bar plot with data points displaying the spread of average ensemble diversity values of the predicted secondary structures in the selected regions. The x axis represents the chromosomes, and the y axis represents their ensemble diversity. Each bar contains 5 dots that correspond to the original ensemble diversity values. The error bars represent the standard deviation.
For (A) and (D), chromosome 4 was not included because it lacks a divergent region, and chromosome X was omitted because it possesses a divergent region <4,000 bp.

prior research highlighting human centromeres to be inherently fragile sites of our genome.[23,26,28]

To accurately measure the impact of complementary base pairing resulting from dyad symmetries on the secondary structures, we calculated the density of dyad symmetries across the selected regions of the centromere and pericentromere (Table S3). We plotted these data for the active HOR, divergent HOR, and centric transition regions to assess the difference between centromeres and pericentromeres. Strikingly, we observed a distinct order of dyad densities, with the centric transition region possessing the highest frequency, followed by the divergent HOR region and the active HOR region (Figures 4A and 4B). This suggests that selective pressure may be working to reduce the number of inverted repeats with large spacer elements that fuel the formation of bulges and loops, which lead to a decrease in overall stability in the active HOR region. This is somewhat surprising because we still observe very high levels of free energy (Figure 1C) and looping hairpins (Figure 2) in the active HOR region. Altogether, our data point to the low self-complementarity of the active centromere sequences as an important factor contributing to DNA secondary-structure complexity. During complementary base pairing, the purine-pyrimidine bond GC/CG is stronger than AT/TA due to the guanine and cytosine being held together by three hydrogen bonds, while only two hydrogen bonds hold adenine and thymine together (Figure 4A).[45] Thus, we assessed the presence of guanines and cytosines in a nucleic acid sequence, its participation in base pairing and base stacking, and how it contributes to increase the stability of the secondary structure. We observed that the centric transition region possesses the highest GC content, followed by the active HOR region and then the divergent HOR region. This can partially explain the MFE values of the secondary structures predicted from the active HOR, divergent HOR, and centric transition regions since the GC contents in all three regions mirror the |MFE| value trends (Figure 4C; Table S4).

Given the presence of inverted repeats did not fully explain the complexity in DNA secondary structures at human centromeres, we evaluated Pearson's correlation coefficient between the MFE values of the predicted secondary structures and the dyad densities evaluated for each region

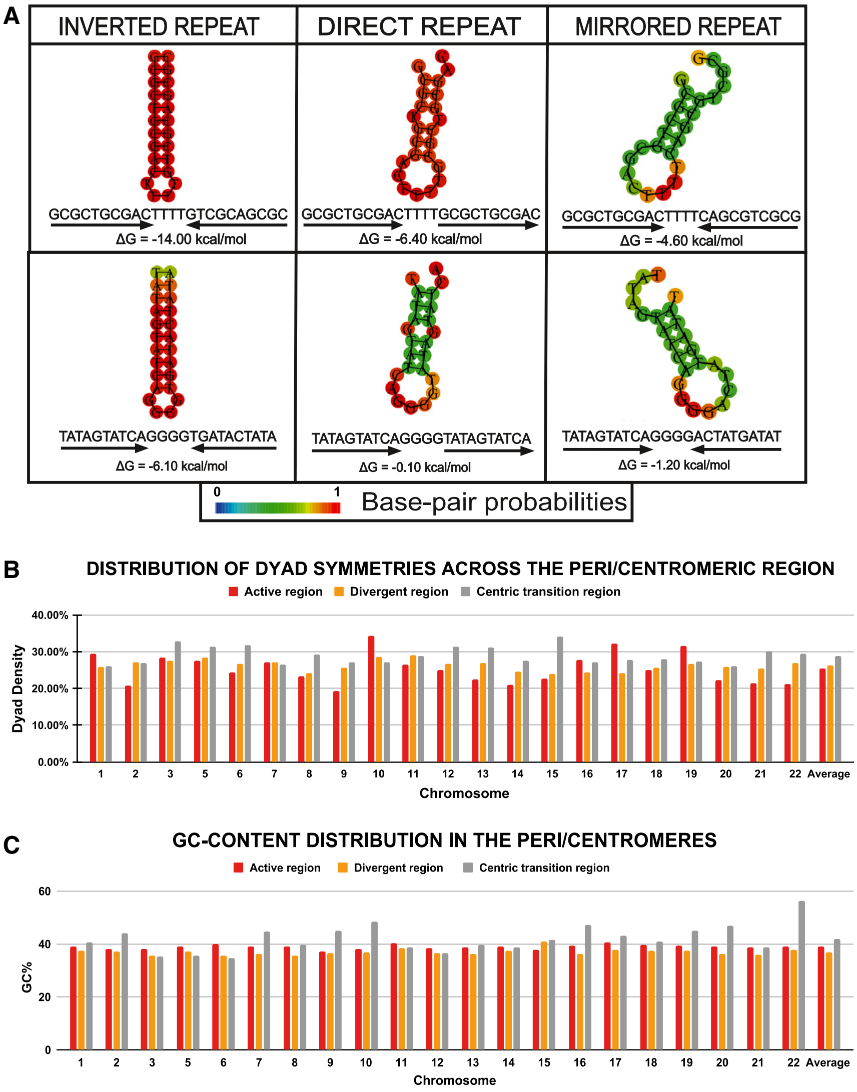

Figure 4. Repeats and their characteristics

(A) An example representing three commonly occurring repeats, inverted, direct, and mirror repeats, along with their predicted secondary structures, as well as the ΔG value. All the repeat sequences in the first row are GC rich and contain the same GC content. The sequences undergo transversion in the second row and are now AT rich. The GC% remains the same in all three sequences in the second row as well. This figure illustrates the secondary structures these repeats form and the differences in their free energy values. The colors represent base pairing probabilities ranging from 0 to 1.

(B) Dyad density distribution in the entirety of the active region, divergent region, and centric transition region of the q arm in all chromosomes.

(C) GC content in the entirety of the active region, divergent region, and centric transition region of the q arm in the chromosomes. Chromosome 4 was omitted because it lacks a divergent region, and chromosome X was omitted because it possesses a divergent region <4,000 bp.

(Figure 5). We used |MFE|, the absolute value of MFE, which directly represents stability as a positive number, in the correlation calculations. Between |MFE| values and dyad densities, a strong positive correlation of r = 0.73 was observed in the active HOR region, a weak positive correlation of r = 0.05 was observed in the divergent HOR region, and surprisingly, a strong negative correlation of r = −0.51 was observed in the centric transition region of the q arms. Because the stem region of a hairpin formed by an inverted repeat contributes to the stability, a negative correlation implies that there are other factors at play that are responsible for the MFE values of the secondary structures. We also included the GC content of the sequence to assess the extent of the influence of sequence composition in determining the free energy value of the secondary structure. The Pearson's correlation coefficients between the |MFE| values and the GC content of the extracted sequences were r = 0.64 in the active HOR region, 0.26 the divergent HOR region, and 0.82 in the centric transition region (Figure 5). These results confirm the trend of the actual MFE values, with the centric transition region showing both the highest GC content and the lowest MFE value, indicating a high level of stability. Similarly, the divergent HOR region possesses the lowest GC content and the highest MFE value, signifying lower stability levels.

Altogether, our observations indicate a complementary relationship between inverted repeats and GC content wherein they contribute to enhance the stabilization of the secondary structure in a synergistic way (Figure 4).

Human centromeres show more complex DNA secondary structures and higher instability than rDNA loci

To get a clearer understanding about the level of complexity of the centromeric repeats, we decided to compare them against another repetitive region in the genome: the rDNA. For the comparative analysis, we selected the acrocentric chromosomes containing rDNA (chr13, chr14, chr15, chr21, and chr22) and extracted five queries of 4,000 bp in length for the following regions: the active HOR, the divergent HOR, the centric transition region, and the rDNA region (Table S1).

The MFE values for the secondary structures predicted from rDNA sequences are more spread out and variable compared to the MFE values of the active and divergent HOR regions (Figure 6A), indicating more variability in their DNA sequences, base pair compositions, and overall secondary structure and implying that the modeling of rDNA copy

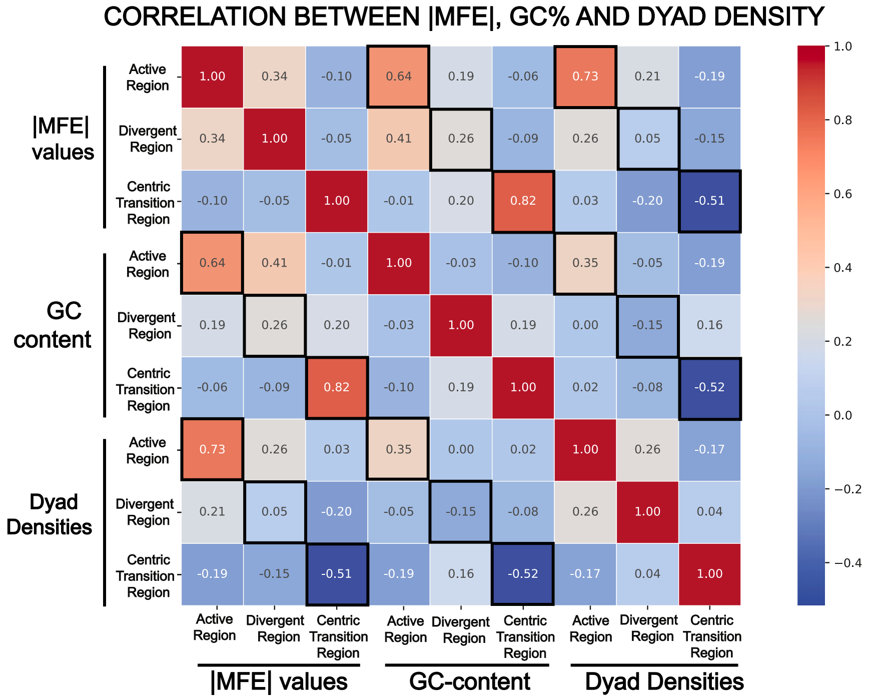

Figure 5. Heatmap showing correlation between the |MFE| values, GC%, and dyad densities across regions: The peri/centromeric active region, divergent region, and centric transition region
|MFE| measurements were utilized in this analysis; therefore, a large positive value of |MFE| corresponds to a secondary structure of higher stability compared to a secondary structure with a relatively smaller positive |MFE| value.

numbers is not the cause of the low complexity. We found the average MFE value of the rDNA secondary structures to be the lowest (Figure 6B), indicating a very high overall thermodynamic stability of the rDNA. Importantly, rDNA still shows lower complexity and higher variability in their MFE value spread compared to the DNA sequences belonging to protein-coding genes. The average MFE was −735.78 kcal/mol for rDNA versus the MFE value of protein-coding genes DNA averaging −490.45 kcal/mol for the acrocentric chromosomes (chr13, chr14, chr15, chr21, and chr22) (Figure 6A). In light of this, it remains striking how the peri-centromeric and centromeric DNA from the active and divergent HOR regions have the highest MFE values and, hence, the highest levels of instability in their secondary structures among all regions considered in this study.

To understand the underlying causes of rDNA kinetics, we assessed the presence of different repeat motifs within ribosomal loci. We found that unlike the peri/centromeric regions, the most commonly occurring motif in rDNA is the mirror repeat (Figure S4A)—a sequence of nucleotides followed downstream by its mirrored sequence (e.g., ATCTCGGC AC CGGCTCTA). The overall occupancy of repeats in the rDNA region follows a roughly homogeneous pattern across the 5 acrocentric chromosomes, suggesting that rDNA is similar in composition across all acrocentric chromosomes or a byproduct of the modeling of the linear sequence in the current CHM13 assembly. We found that the rDNA sequences contain a substantially greater amount of GC content compared to the peri/centromeric regions (Figure S4B). The high GC content in the rDNA region likely plays a pivotal role in the MFE values observed in the secondary structures. Overall, this implies a high level of stability in the rDNA region and confirms the notion that centromere

DNA is one of the most complex and dynamically unstable loci in our genome, even when compared with other repetitive regions.

Complexity in predicted secondary structures at centromeres in CHM13 and RPE1 reference genomes correlates with chromosomes' missegregation rates

Previous studies of DNA secondary structures have assessed multiple species, including human GRCh38, primates, mouse, and yeast genomes.[18–20] Our work provides a resource of secondary-structure prediction based on the newly assembled telomere-to-telomere (T2T) CHM13 human genome, including complete regions spanning repetitive satellite DNA. Next, we wanted to understand whether our finding using "sequence-based" topological prediction to reveal the complexity and instability of centromeres may have functional and biological significance and impact chromosome segregation. To address this question, we used our chromosome-specific measurements derived in this study and correlated them to the rates of missegregation previously calculated for each human chromosome.[46] We found that the rate of chromosome missegregation is directly correlated with low |MFE| values, indicating the high complexity of secondary structures (Figure 7A; Table S1). The estimates for chromosome-specific missegregation rates were calculated through experiments performed using the retinal epithelial diploid cell line RPE-1. Because our laboratory recently assembled the T2T reference genome for RPE-1 (RPE1v1.0),[9] we compared the RPE-1 chromosome-specific missegregation rates with the MFE values estimated using 1,000 bp of DNA sequence from the centromeres in the RPE1v1.0 assembly[9] (Table S6). Strikingly, in both RPE-1 haplotype 1 and haplotype 2 (Figures S6A and S6B), the chromosome-specific |MFE| value inversely correlated with the propensity of missegregation of the specific chromosome in RPE-1. These data indicate that low |MFE| values, indicating high complexity and instability in DNA secondary structures, may affect the fidelity of centromere function and, in turn, influence chromosome dynamics and their faithful segregation into daughter cells. This is particularly

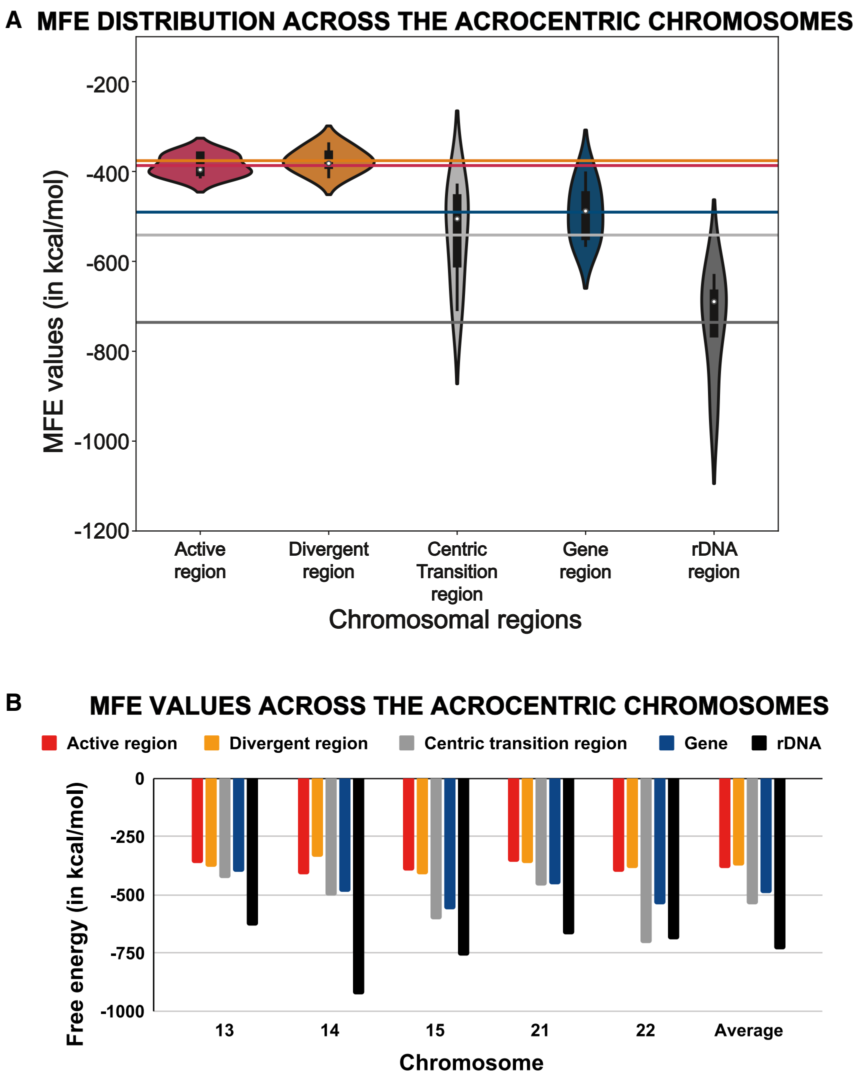

Figure 6. Centromeric MFE value comparison against the rDNA region in the acrocentric chromosomes
(A) Violin plot displaying the distribution of the MFE values in the acrocentric chromosomes across the active region, divergent region, centric transition region, gene region, and rDNA region. The horizontal lines correspond to the overall mean for each region.
(B) MFE value comparison of the five aforementioned regions across all acrocentric chromosomes.

interesting, as the rates of missegregation were calculated both in untreated cells and upon disruption of CENP-A chromatin,[46] which could provide a direct *in vivo* opportunity for linear DNA topology to undergo intra-strand complementarity and form secondary structures of high complexity with low |MFE| values (Figure 7B), causing aneuploidy.[23]

Discussion

Here, we analyzed linear DNA sequences for key regions of our chromosomes: from the center, we sampled the active HOR of the centromeres, divergent arrays, the pericentromere transition region, the gene regions along the chromosome's arms, and finally, the rDNA of acrocentric chromosomes. We provide an annotated table with the linear information for all chromosome studies (Table S1) and all code used in this study (https://github.com/GiuntaLab/DNA-secondary-structures-analysis). Due to the lack of a complete linear annotation of repetitive regions like the centromeres in previous human genome references, our study offers a resource for a variety of genomic analyses pertaining to these human loci. Here, we looked at DNA secondary-structure prediction and found the highest DNA dynamics given by the free energy value in centromeric loci. Secondary structures predicted from centromeric DNA exhibit relatively homogeneous free energy distribution across all chromosomes, likely indicating a maximum level of tolerance for a functional locus. The secondary structures predicted from pericentromeric DNA also display high complexity, with a level of heterogeneity among the values of free energy that likely reflects the more varied sequence composition made of diverging alpha-satellite monomers as well as other repeats. Importantly, our trends are conserved when using DNA sequences of 500 bp and 1 kb compared to our 4 kb results, showing that the length—while changing the actual MFE value—does not artificially influence the MFE trend we calculated, which held true for all sizes of DNA analyzed across all regions sampled (Table S5; Figures S5A–S5C). Sampling three different sizes in 5 different spots for each region also gave us an opportunity to assess predicted secondary structures of single-stranded DNA emerging from different biologically relevant mechanisms. For instance, break-induced dechromatinization may expose a shorter stretch of naked DNA, while lncRNA transcription can expose longer stretches over 1 kb in length.

The process of calculating the MFE value of a secondary structure involves a complex interplay among multiple factors. One of the most used computational approaches for the evaluation of the MFE value of a DNA/RNA secondary structure is the nearest-neighbor method. This approach considers the energy contributions of every base pair and their interactions with the neighboring base pair and loop regions. Therefore, assessing the MFE

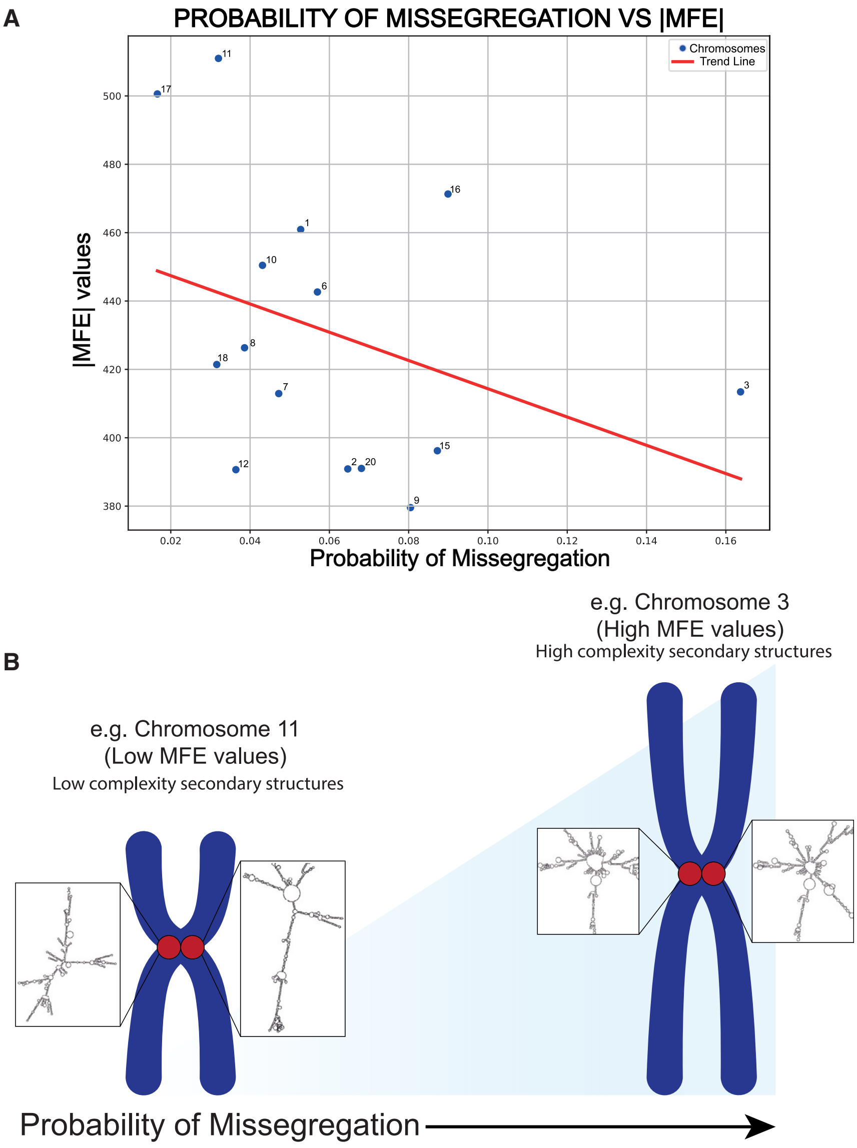

Figure 7. Relationship between MFE values and the probability of missegregation

(A) A scatterplot with the probability of missegregation on the x axis[46] and the |MFE| values of the secondary structures predicted for the sequences on the y axis from the active region of chromosomes from CHM13 assembly. We observed a correlation of −0.36 between the absolute MFE values and the probability of missegregation. The data used to obtain this graph are available in Table S5.

(B) An illustration depicting the correlation between the actual MFE values and the probability of missegregation. Predicted secondary structures from the chromosome 11 centromere (two examples shown in the zoom in) are relatively stable and possess low MFE values and, hence, are associated with a lower probability of missegregation; on the other hand, chromosome 3 DNA sequences show folding into complex secondary structures (two examples shown in the zoom in) with high MFE values and are associated with a higher propensity for missegregation.

values, as well as studying the complexity of the predicted secondary structures, provides great insights regarding the nature and characteristics of a given chromosomal region. One factor playing a major role in the MFE value determination of a secondary structure is the size, type, and frequency of occurrence of the loops in it. A possible cause for the high MFE value in the centromeres' active and divergent regions could be attributed to the presence of complementary repeats building topological structures harboring short stems and large stem loops, hence leading to an overall increase in the MFE value. The presence of DNA secondary structures and the ensuing instability could mechanistically underlie the phenomenon observed in the centromeric evolution model of layered expansion. According to this model, the active HOR region undergoes insertions, deletions, and high levels of mutagenesis. To maintain function, a new active HOR is formed, pushing the older, mutated DNA to the side as it becomes more divergent. This hypothesis seems to fit with the recent linear assembly of centromere DNA for most human chromosomes.[8] Centromeres are organized with the active HOR in the center, flanked by the neighboring inactive HOR and divergent HOR regions. Divergent HOR represents a probable evolutionary relic of the region that previously was the active HOR before the occurrence of layered expansion. This is also supported by the notion that centromeres are rapidly evolving regions in the human genome.[47,48] Our data are in line with this hypothesis, as we observed that the divergent HOR region, when compared to the active HOR region, possessed a lower GC content but a higher dyad density and, ultimately, was predicted to form complex secondary structures due to a slightly higher MFE value compared to the active HOR region. It is possible that, as centromeres mutagenize—which happens at a higher rate than other regions, as we and others have shown[26,48]—the instability of DNA and the complexity of the secondary structure become functionally disruptive and need to be replaced by fresh expansion of HORs, serving as the kinetochore site to maintain chromosome segregation. Indeed, despite their physical proximity, the MFE values of the secondary structures predicted in the divergent HOR region, while remarkably close, are slightly higher (i.e., less stable) than those predicted in the active HOR region. This disparity *in vivo* can be further altered by protein-binding and CENP-A interactions present in the active HOR region, which are

essential for kinetochore assembly, whereas the divergent region lacks these features.[8]

The mirrored repeats found in high quantities in the rDNA (Figure S4A) have not been shown to be associated with specific non-B DNA structures.[49] Accordingly, we found less higher-order structures compared to alpha-satellite repeats. This may be attributed to the functional significance of rDNA or the modeling of the rDNA sequence based on the copy number. While the secondary structures depicted in this analysis are predictions, and we cannot be certain that such structures exist as per our simulations and/or how the nuclear, chromatin, and molecular environment influences their actual configurations, there are possibilities for their formation *in vivo*. rDNA undergoes transcription to give rise to rRNA, which is an essential component of the protein biosynthesis complex, the ribosome.[50] Similarly, centromeres show low levels of transcription into kilobase-long ncRNAs.[36] Thus, transcription, R-loops, repair, and other DNA-based transactions offer opportunities for self-hybridization. Our finding is in line with previous studies among multiple species.[18–20] Accordingly, when we used the T2T diploid reference genome for the laboratory cell RPE-1 assembled by our laboratory,[9] we found the same trend for MFE values with high centromere complexity in predicted DNA topology, implying that secondary structures are indeed intrinsic properties of centromeres. To this end, our chromosome-specific measurements correlate with the probability of missegregation calculated for human chromosomes. We found that an increased propensity for the specific chromosome to missegregate correlates with low |MFE| values, indicating the presence of high complexity of secondary structures within that centromere (Figure 7A). The correlation held true in CHM13 (Figures 7A and 7B), as well as when using the reference genome for RPE-1 that our laboratory recently generated,[9] for both haplotypes (Figures S6A and S6B). These data indicate that low |MFE| values and high complexity and instability in DNA secondary structures may affect the fidelity of centromere function and, in turn, influence chromosome dynamics and their faithful segregation into daughter cells. This is particularly interesting, as the estimated chromosome-specific missegregation rates were calculated through experiments performed using the retinal epithelial diploid cell line RPE-1, enabling a more direct comparison using the recently assembled RPE1v1.0 genome.[9] Furthermore, the missegregation was evaluated upon disruption of CENP-A chromatin,[46] which could provide a direct *in vivo* opportunity for linear DNA topology to undergo intra-strand complementarity and form the high-complexity secondary structures centromeres are capable of (Figure 7B).

Given the rapidly changing nature and variability of these repetitive loci across evolution and in organisms from the same species, there is huge potential for secondary structures to impact the evolutionary course of an organism's genome.[51] Our work offers a viewpoint on centromeres and other genomic loci while considering the secondary structures to gain a multilayered understanding of their function. A compromised centromere function can lead to nondisjunction, a major cause of somatic and germline diseases.[52] DNA secondary structures also play an important role in recognizing proteins as well as defining the origin of replication in many single-stranded DNA viruses.[53,54] DNA secondary structures also play an integral role in the survival and propagation of retroviruses, as they participate in activities like strand jumping.[55] Several biotechnology techniques that exploit the three-dimensional folding potential of DNA have been demonstrated, including DNA nanotechnology[56] and DNA computing.[57] Taking these results together, we highlight the pivotal significance of studying DNA secondary structures in understanding human genomic loci in light of their functional topology.

Data and code availability

All the datasets generated and analyzed during this study are available in the supplemental information. The code associated with this study is available in our lab Github: https://github.com/GiuntaLab/DNA-secondary-structures-analysis.

Acknowledgments

We thank Valentina Liguori and all members of the Giunta lab for helpful discussions. We thank Luca Corda for critically reading the manuscript and providing feedback, Evelyne Tassone for assistance with the reference list, Elena Di Tommaso for key help with the cartoon in Figure 7, and Professor Valerio Fulci for helpful insights into energy value calculations for DNA sequestration from inter-strand complementarity. All computing was possible thanks to Professor Umberto Ferraro Petrillo and CINECA HPC and Terastat2 Sapienza Server. This work was funded thanks to the Italian Foundation for Cancer Research (AIRC Start-Up Grant 2020 ID# 25189) and Sapienza University of Rome DR no. 525-2024 "Principal Investigator Project of Excellence" funding.

Declaration of interests

The authors declare no competing interests.

Supplemental information

Supplemental information can be found online at https://doi.org/10.1016/j.ajhg.2024.10.016.

Received: April 18, 2024
Accepted: October 25, 2024
Published: November 18, 2024

References

1. McKinley, K.L., and Cheeseman, I.M. (2016). The molecular basis for centromere identity and function. Nat. Rev. Mol. Cell Biol. *17*, 16–29. https://doi.org/10.1038/nrm.2015.5.
2. Musacchio, A., and Desai, A. (2017). A Molecular View of Kinetochore Assembly and Function. Biology *6*, 5. https://doi.org/10.3390/biology6010005.

3. De Bustos, A., Cuadrado, A., and Jouve, N. (2016). Sequencing of long stretches of repetitive DNA. Sci. Rep. *6*, 36665. https://doi.org/10.1038/srep36665.
4. Treangen, T.J., and Salzberg, S.L. (2011). Repetitive DNA and next-generation sequencing: computational challenges and solutions. Nat. Rev. Genet. *13*, 36–46. https://doi.org/10.1038/nrg3117.
5. Morton, E.A., Hall, A.N., Kwan, E., Mok, C., Queitsch, K., Nandakumar, V., Stamatoyannopoulos, J., Brewer, B.J., Waterston, R., and Queitsch, C. (2020). Challenges and Approaches to Genotyping Repetitive DNA. G3 (Bethesda) *10*, 417–430. https://doi.org/10.1534/g3.119.400771.
6. Nurk, S., Koren, S., Rhie, A., Rautiainen, M., Bzikadze, A.V., Mikheenko, A., Vollger, M.R., Altemose, N., Uralsky, L., Gershman, A., et al. (2022). The complete sequence of a human genome. Science *376*, 44–53. https://doi.org/10.1126/science.abj6987.
7. Lander, E.S., Linton, L.M., Birren, B., Nusbaum, C., Zody, M.C., Baldwin, J., Devon, K., Dewar, K., Doyle, M., FitzHugh, W., et al. (2001). Initial sequencing and analysis of the human genome. Nature *409*, 860–921. https://doi.org/10.1038/35057062.
8. Altemose, N., Logsdon, G.A., Bzikadze, A.V., Sidhwani, P., Langley, S.A., Caldas, G.V., Hoyt, S.J., Uralsky, L., Ryabov, F.D., Shew, C.J., et al. (2022). Complete genomic and epigenetic maps of human centromeres. Science *376*, eabl4178. https://doi.org/10.1126/science.abl4178.
9. Volpe, E., Corda, L., Di Tommaso, E., Pelliccia, F., Ottalevi, R., Licastro, D., Guarracino, A., Capulli, M., Formenti, G., Tassone, E., and Giunta, S. (2023). The complete diploid reference genome of RPE-1 identifies human phased epigenetic landscapes. Preprint at bioRxiv. https://doi.org/10.1101/2023.11.01.565049.
10. McNulty, S.M., and Sullivan, B.A. (2018). Alpha satellite DNA biology: finding function in the recesses of the genome. Chromosome Res. *26*, 115–138. https://doi.org/10.1007/s10577-018-9582-3.
11. Fachinetti, D., Folco, H.D., Nechemia-Arbely, Y., Valente, L.P., Nguyen, K., Wong, A.J., Zhu, Q., Holland, A.J., Desai, A., Jansen, L.E.T., and Cleveland, D.W. (2013). A two-step mechanism for epigenetic specification of centromere identity and function. Nat. Cell Biol. *15*, 1056–1066. https://doi.org/10.1038/ncb2805.
12. Watson, J.D., and Crick, F.H. (1953). Molecular structure of nucleic acids; a structure for deoxyribose nucleic acid. Nature *171*, 737–738. https://doi.org/10.1038/171737a0.
13. Franklin, R.E., and Gosling, R.G. (1953). Molecular configuration in sodium thymonucleate. Nature *171*, 740–741. https://doi.org/10.1038/171740a0.
14. Tinoco, I., and Bustamante, C. (1999). How RNA folds. J. Mol. Biol. *293*, 271–281. https://doi.org/10.1006/jmbi.1999.3001.
15. Tinoco, I., Uhlenbeck, O.C., and Levine, M.D. (1971). Estimation of secondary structure in ribonucleic acids. Nature *230*, 362–367. https://doi.org/10.1038/230362a0.
16. Matos-Rodrigues, G., Hisey, J.A., Nussenzweig, A., and Mirkin, S.M. (2023). Detection of alternative DNA structures and its implications for human disease. Mol. Cell *83*, 3622–3641. https://doi.org/10.1016/j.molcel.2023.08.018.
17. Bansal, A., Kaushik, S., and Kukreti, S. (2022). Non-canonical DNA structures: Diversity and disease association. Front. Genet. *13*, 959258. https://doi.org/10.3389/fgene.2022.959258.
18. Kasinathan, S., and Henikoff, S. (2018). Non-B-Form DNA Is Enriched at Centromeres. Mol. Biol. Evol. *35*, 949–962. https://doi.org/10.1093/molbev/msy010.
19. Patchigolla, V.S.P., and Mellone, B.G. (2022). Enrichment of Non-B-Form DNA at D. melanogaster Centromeres. Genome Biol. Evol. *14*, evac054. https://doi.org/10.1093/gbe/evac054.
20. Liu, Q., Yi, C., Zhang, Z., Su, H., Liu, C., Huang, Y., Li, W., Hu, X., Liu, C., Birchler, J.A., et al. (2023). Non-B-form DNA tends to form in centromeric regions and has undergone changes in polyploid oat subgenomes. Proc. Natl. Acad. Sci. USA *120*, e2211683120. https://doi.org/10.1073/pnas.2211683120.
21. Weissensteiner, M.H., Cremona, M.A., Guiblet, W.M., Stoler, N., Harris, R.S., Cechova, M., Eckert, K.A., Chiaromonte, F., Huang, Y.F., and Makova, K.D. (2023). Accurate sequencing of DNA motifs able to form alternative (non-B) structures. Genome Res. *33*, 907–922. https://doi.org/10.1101/gr.277490.122.
22. Rhie, A., Nurk, S., Cechova, M., Hoyt, S.J., Taylor, D.J., Altemose, N., Hook, P.W., Koren, S., Rautiainen, M., Alexandrov, I.A., et al. (2023). The complete sequence of a human Y chromosome. Nature *621*, 344–354. https://doi.org/10.1038/s41586-023-06457-y.
23. Giunta, S., Hervé, S., White, R.R., Wilhelm, T., Dumont, M., Scelfo, A., Gamba, R., Wong, C.K., Rancati, G., Smogorzewska, A., et al. (2021). CENP-A chromatin prevents replication stress at centromeres to avoid structural aneuploidy. Proc. Natl. Acad. Sci. USA *118*, e2015634118. https://doi.org/10.1073/pnas.2015634118.
24. Jonstrup, A.T., Thomsen, T., Wang, Y., Knudsen, B.R., Koch, J., and Andersen, A.H. (2008). Hairpin structures formed by alpha satellite DNA of human centromeres are cleaved by human topoisomerase IIα. Nucleic Acids Res. *36*, 6165–6174. https://doi.org/10.1093/nar/gkn640.
25. Ohno, M., Fukagawa, T., Lee, J.S., and Ikemura, T. (2002). Triplex-forming DNAs in the human interphase nucleus visualized in situ by polypurine/polypyrimidine DNA probes and antitriplex antibodies. Chromosoma *111*, 201–213. https://doi.org/10.1007/s00412-002-0198-0.
26. Black, E.M., and Giunta, S. (2018). Repetitive Fragile Sites: Centromere Satellite DNA as a Source of Genome Instability in Human Diseases. Genes *9*, 615. https://doi.org/10.3390/genes9120615.
27. Maccaroni, K., Balzano, E., Mirimao, F., Giunta, S., and Pelliccia, F. (2020). Impaired Replication Timing Promotes Tissue-Specific Expression of Common Fragile Sites. Genes *11*, 326. https://doi.org/10.3390/genes11030326.
28. Balzano, E., Pelliccia, F., and Giunta, S. (2021). Genome (in) stability at tandem repeats. Semin. Cell Dev. Biol. *113*, 97–112. https://doi.org/10.1016/j.semcdb.2020.10.003.
29. Giunta, S., and Funabiki, H. (2017). Integrity of the human centromere DNA repeats is protected by CENP-A, CENP-C, and CENP-T. Proc. Natl. Acad. Sci. USA *114*, 1928–1933. https://doi.org/10.1073/pnas.1615133114.
30. Lorenz, R., Bernhart, S.H., Höner Zu Siederdissen, C., Tafer, H., Flamm, C., Stadler, P.F., and Hofacker, I.L. (2011). ViennaRNA Package 2.0. Algorithms. Mol Biol *6*, 26. https://doi.org/10.1186/1748-7188-6-26.
31. Zuker, M., and Stiegler, P. (1981). Optimal computer folding of large RNA sequences using thermodynamics and auxiliary information. Nucleic Acids Res. *9*, 133–148.
32. Turner, D.H., and Mathews, D.H. (2010). NNDB: the nearest neighbor parameter database for predicting stability of nucleic

acid secondary structure. Nucleic Acids Res. *38*, D280–D282. https://doi.org/10.1093/nar/gkp892.

33. SantaLucia, J. (1998). A unified view of polymer, dumbbell, and oligonucleotide DNA nearest-neighbor thermodynamics. Proc. Natl. Acad. Sci. USA *95*, 1460–1465.
34. Laing, C., and Schlick, T. (2010). Computational approaches to 3D modeling of RNA. J. Phys. Condens. Matter *22*, 283101. https://doi.org/10.1088/0953-8984/22/28/283101.
35. Kerpedjiev, P., Hammer, S., and Hofacker, I.L. (2015). Forna (force-directed RNA): Simple and effective online RNA secondary structure diagrams. Bioinformatics *31*, 3377–3379. https://doi.org/10.1093/bioinformatics/btv372.
36. Bury, L., Moodie, B., Ly, J., McKay, L.S., Miga, K.H., and Cheeseman, I.M. (2020). Alpha-satellite RNA transcripts are repressed by centromere-nucleolus associations. Elife *9*, e59770. https://doi.org/10.7554/eLife.59770.
37. Cer, R.Z., Bruce, K.H., Donohue, D.E., Temiz, N.A., Mudunuri, U.S., Yi, M., Volfovsky, N., Bacolla, A., Luke, B.T., Collins, J.R., and Stephens, R.M. (2012). Searching for non-B DNA-forming motifs using nBMST (non-B DNA Motif Search Tool). Curr. Protoc. Hum. Genet. *Chapter 18*, 722–18.7.22. CHAPTER:Unit-18. https://doi.org/10.1002/0471142905.hg1807s73.
38. Rice, P., Longden, I., and Bleasby, A. (2000). EMBOSS: the European Molecular Biology Open Software Suite. Trends Genet. *16*, 276–277. https://doi.org/10.1016/s0168-9525(00)02024-2.
39. Di Tommaso, E., de Turris, V., Choppakatla, P., Funabiki, H., and Giunta, S. (2023). Visualization of the three-dimensional structure of the human centromere in mitotic chromosomes by superresolution microscopy. Mol. Biol. Cell *34*, ar61. https://doi.org/10.1091/mbc.E22-08-0332.
40. Gibbs, J.W., Josiah, W., and Tyndall, J. (1874). Connecticut Academy of Arts and Sciences, Burndy Library Donor D. On the Equilibrium of Heterogeneous Substances : First [-second] Part (New Haven : Published by the Academy).
41. Moulton, V., Zuker, M., Steel, M., Pointon, R., and Penny, D. (2000). Metrics on RNA secondary structures. J. Comput. Biol. *7*, 277–292. https://doi.org/10.1089/10665270050081522.
42. Freyhult, E., Gardner, P.P., and Moulton, V. (2005). A comparison of RNA folding measures. BMC Bioinf. *6*, 241. https://doi.org/10.1186/1471-2105-6-241.
43. Hallast, P., Ebert, P., Loftus, M., Yilmaz, F., Audano, P.A., Logsdon, G.A., Bonder, M.J., Zhou, W., Höps, W., Kim, K., et al. (2023). Assembly of 43 human Y chromosomes reveals extensive complexity and variation. Nature *621*, 355–364. https://doi.org/10.1038/s41586-023-06425-6.
44. Lu, S., Wang, G., Bacolla, A., Zhao, J., Spitser, S., and Vasquez, K.M. (2015). Short Inverted Repeats Are Hot Spots For Genetic Instability: Relevance to Cancer Genomes. Cell Rep. *10*, 1674–1680. https://doi.org/10.1016/j.celrep.2015.02.039.
45. Yakovchuk, P., Protozanova, E., and Frank-Kamenetskii, M.D. (2006). Base-stacking and base-pairing contributions into thermal stability of the DNA double helix. Nucleic Acids Res. *34*, 564–574. https://doi.org/10.1093/nar/gkj454.
46. Dumont, M., Gamba, R., Gestraud, P., Klaasen, S., Worrall, J.T., De Vries, S.G., Boudreau, V., Salinas-Luypaert, C., Maddox, P.S., Lens, S.M., et al. (2020). Human chromosome-specific aneuploidy is influenced by DNA-dependent centromeric features. EMBO J. *39*, e102924. https://doi.org/10.15252/embj.2019102924.
47. Henikoff, S., and Malik, H.S. (2002). Centromeres: selfish drivers. Nature *417*, 227. https://doi.org/10.1038/417227a.
48. Logsdon, G.A., and Eichler, E.E. (2022). The Dynamic Structure and Rapid Evolution of Human Centromeric Satellite DNA. Genes *14*, 92. https://doi.org/10.3390/genes14010092.
49. Cer, R.Z., Bruce, K.H., Mudunuri, U.S., Yi, M., Volfovsky, N., Luke, B.T., Bacolla, A., Collins, J.R., and Stephens, R.M. (2011). Non-B DB: a database of predicted non-B DNA-forming motifs in mammalian genomes. Nucleic Acids Res. *39*, D383–D391. https://doi.org/10.1093/nar/gkq1170.
50. Srivastava, A.K., and Schlessinger, D. (1991). Structure and organization of ribosomal DNA. Biochimie *73*, 631–638. https://doi.org/10.1016/0300-9084(91)90042-y.
51. Guiblet, W.M., Cremona, M.A., Harris, R.S., Chen, D., Eckert, K.A., Chiaromonte, F., Huang, Y.F., and Makova, K.D. (2021). Non-B DNA: a major contributor to small- and large-scale variation in nucleotide substitution frequencies across the genome. Nucleic Acids Res. *49*, 1497–1516. https://doi.org/10.1093/nar/gkaa1269.
52. Nagaoka, S.I., Hassold, T.J., and Hunt, P.A. (2012). Human aneuploidy: mechanisms and new insights into an age-old problem. Nat. Rev. Genet. *13*, 493–504. https://doi.org/10.1038/nrg3245.
53. Ikoku, A.S., and Hearst, J.E. (1981). Identification of a structural hairpin in the filamentous chimeric phage M13Gori1. J. Mol. Biol. *151*, 245–259. https://doi.org/10.1016/0022-2836(81)90514-3.
54. Russel, M. (1994). Mutants at conserved positions in gene IV, a gene required for assembly and secretion of filamentous phages. Mol. Microbiol. *14*, 357–369. https://doi.org/10.1111/j.1365-2958.1994.tb01296.x.
55. Buiser, R.G., DeStefano, J.J., Mallaber, L.M., Fay, P.J., and Bambara, R.A. (1991). Requirements for the catalysis of strand transfer synthesis by retroviral DNA polymerases. J. Biol. Chem. *266*, 13103–13109.
56. Seeman, N.C. (2003). Biochemistry and structural DNA nanotechnology: an evolving symbiotic relationship. Biochemistry *42*, 7259–7269. https://doi.org/10.1021/bi030079v.
57. Gibbons, A., Amos, M., and Hodgson, D. (1997). DNA computing. Curr. Opin. Biotechnol. *8*, 103–106. https://doi.org/10.1016/s0958-1669(97)80164-4.

The American Journal of Human Genetics, Volume *111*

Supplemental information

Comparative analysis of predicted DNA secondary structures infers complex human centromere topology

Sai Swaroop Chittoor and Simona Giunta

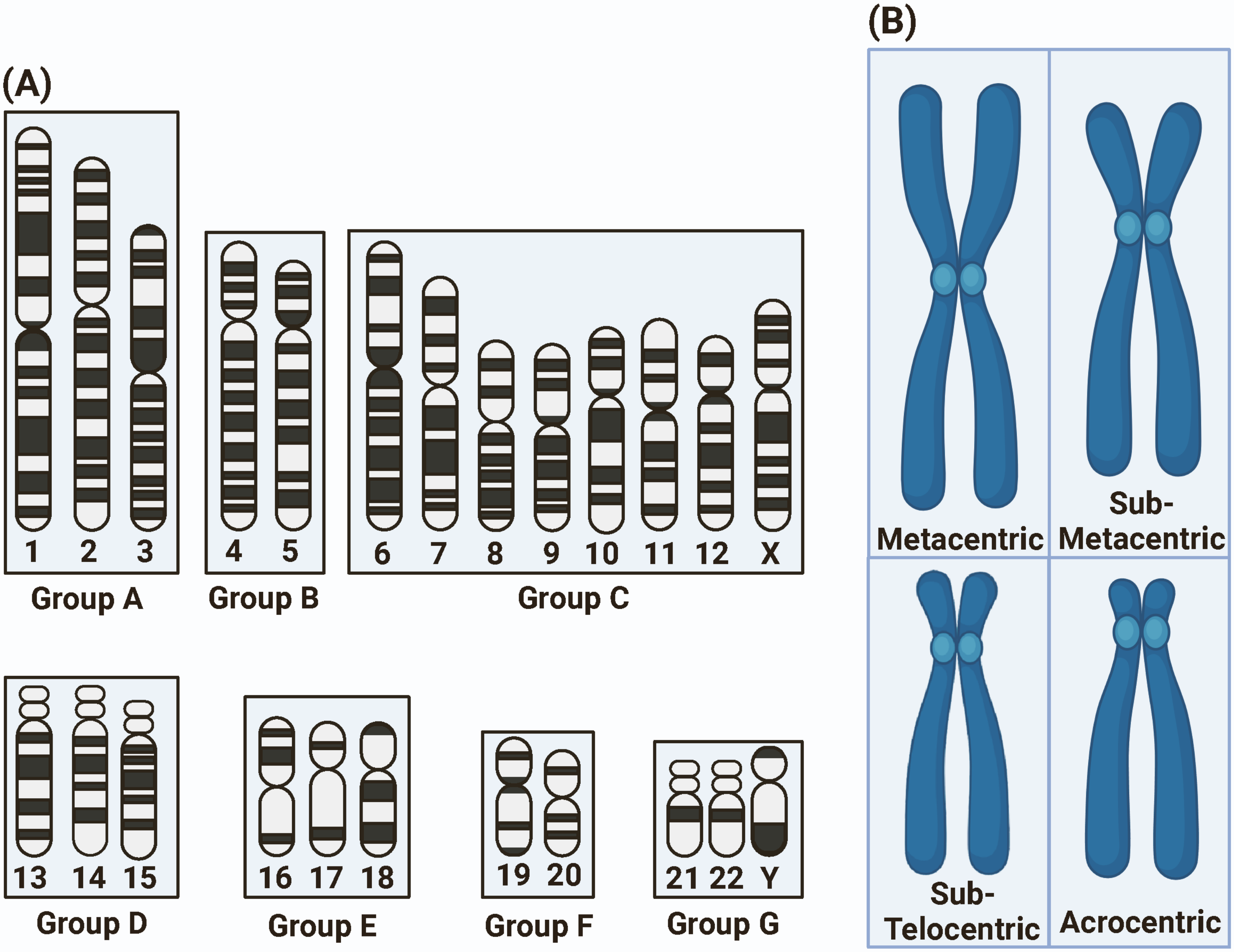
(A)
1
2
3
Group A
4
5
Group B
6
7
8
9
10
11
12
X
Group C
13
14
15
Group D
16
17
18
Group E
19
20
Group F
21
22
Y
Group G
(B)
Metacentric
Sub-Metacentric
Sub-Telocentric
Acrocentric

FIGURE S1

Figure S1. Classification of chromosomes based on centromere position. (A) Group-wise classification of 22+2 chromosomes in the human karyotype. Group A contains chromosomes 1, 2 and 3. These chromosomes are the largest and are metacentric with a centrally positioned centromere with two equivalent arms. Group B consists of chromosomes 4 and 5 which are submetacentric. Group C is the one with the largest number of chromosomes. It consists of chromosomes 6-12 and chromosome X. Group D comprises acrocentric chromosomes - 13,14 and 15. Group E contains chromosomes 16, 17 and 18, which are short meta/submetacentric chromosomes. Group F consists of short metacentric chromosomes 19 and 20 and finally Group G comprises very short acrocentric chromosomes 21, 22 and chromosome Y. (B) Four distinct types of chromosomes based on the centromere position. The figure was created using BioRender (2020).

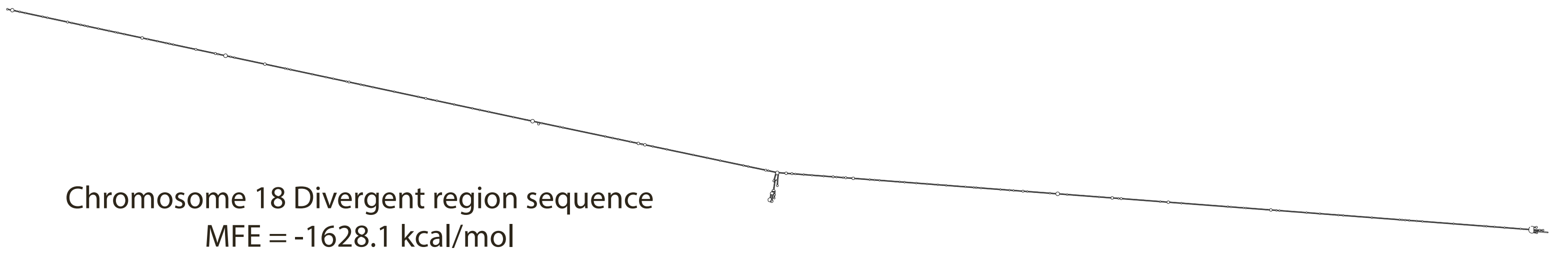
Chromosome 18 Divergent region sequence
MFE = -1628.1 kcal/mol

FIGURE S2

Figure S2. Secondary structure prediction of a DNA sequence from the divergent region of chromosome 18. Despite the sequence belonging to the divergent region, this secondary structure is the most stable with the lowest MFE value.

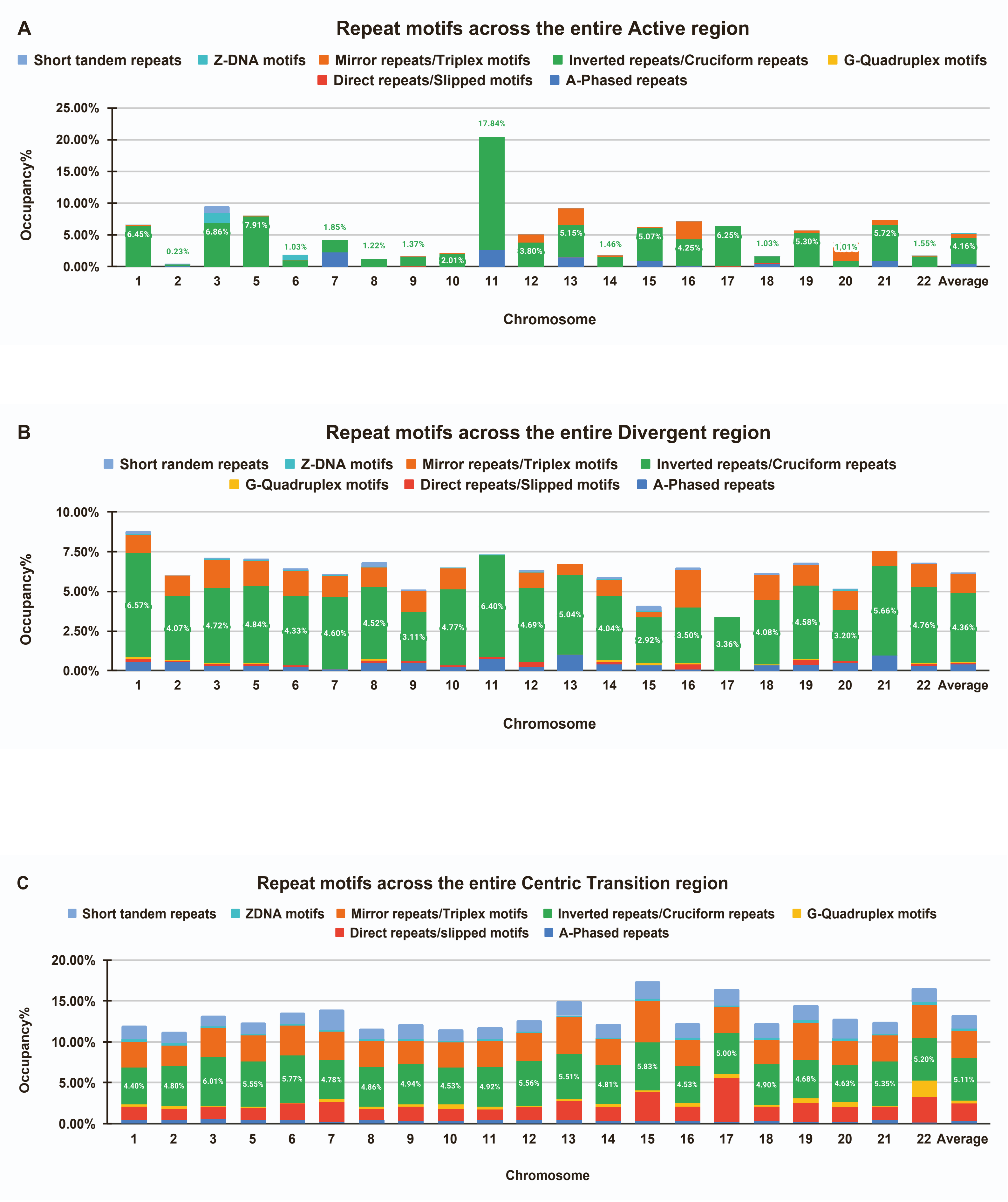
A
Repeat motifs across the entire Active region
Short tandem repeats
Z-DNA motifs
Mirror repeats/Triplex motifs
Inverted repeats/Cruciform repeats
G-Quadruplex motifs
Direct repeats/Slipped motifs
A-Phased repeats
Occupancy%
25.00%
20.00%
15.00%
10.00%
5.00%
0.00%
6.45%
0.23%
6.86%
7.91%
1.03%
1.85%
1.22%
1.37%
2.01%
17.84%
3.80%
5.15%
1.46%
5.07%
4.25%
6.25%
1.03%
5.30%
1.01%
5.72%
1.55%
4.16%
1 2 3 5 6 7 8 9 10 11 12 13 14 15 16 17 18 19 20 21 22 Average
Chromosome
B
Repeat motifs across the entire Divergent region
Short randem repeats
Z-DNA motifs
Mirror repeats/Triplex motifs
Inverted repeats/Cruciform repeats
G-Quadruplex motifs
Direct repeats/Slipped motifs
A-Phased repeats
Occupancy%
10.00%
7.50%
5.00%
2.50%
0.00%
6.57%
4.07%
4.72%
4.84%
4.33%
4.60%
4.52%
3.11%
4.77%
6.40%
4.69%
5.04%
4.04%
2.92%
3.50%
3.36%
4.08%
4.58%
3.20%
5.66%
4.76%
4.36%
1 2 3 5 6 7 8 9 10 11 12 13 14 15 16 17 18 19 20 21 22 Average
Chromosome
C
Repeat motifs across the entire Centric Transition region
Short tandem repeats
ZDNA motifs
Mirror repeats/Triplex motifs
Inverted repeats/Cruciform repeats
G-Quadruplex motifs
Direct repeats/slipped motifs
A-Phased repeats
Occupancy%
20.00%
15.00%
10.00%
5.00%
0.00%
4.40%
4.80%
6.01%
5.55%
5.77%
4.78%
4.86%
4.94%
4.53%
4.92%
5.56%
5.51%
4.81%
5.83%
4.53%
5.00%
4.90%
4.68%
4.63%
5.35%
5.20%
5.11%
1 2 3 5 6 7 8 9 10 11 12 13 14 15 16 17 18 19 20 21 22 Average
Chromosome

FIGURE S3

Figure S3. Repeat motif occupancy. Occupancy% of short tandem repeats, Z-DNA motifs, mirror repeats, inverted repeats, G4 motifs, Direct repeats and A-Phased repeats in the entirety of the (A) Active region, (B) Divergent region and (C) Centric Transition region.

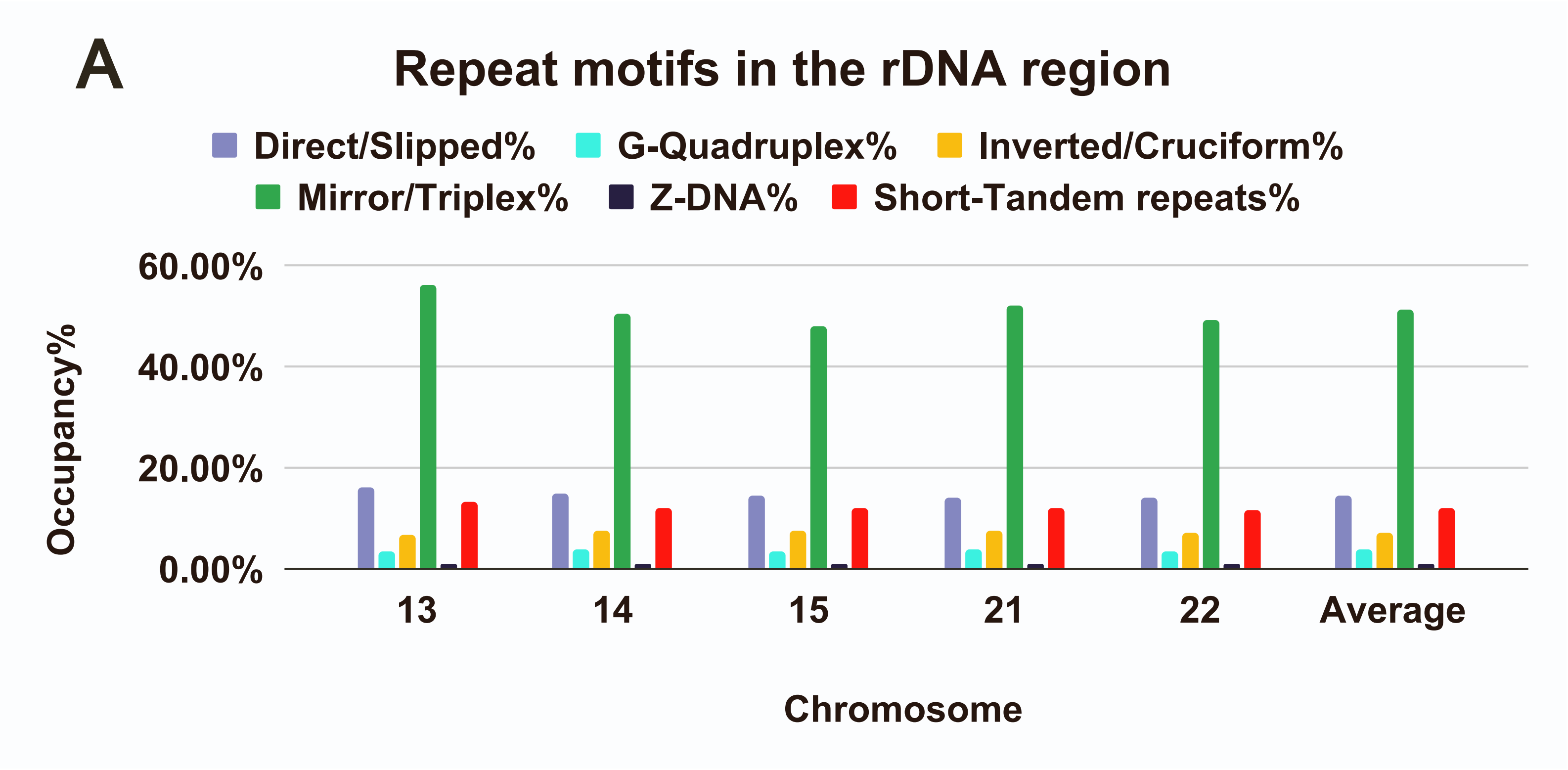
A
Repeat motifs in the rDNA region
Direct/Slipped%
G-Quadruplex%
Inverted/Cruciform%
Mirror/Triplex%
Z-DNA%
Short-Tandem repeats%
60.00%
40.00%
20.00%
0.00%
Occupancy%
13
14
15
21
22
Average
Chromosome

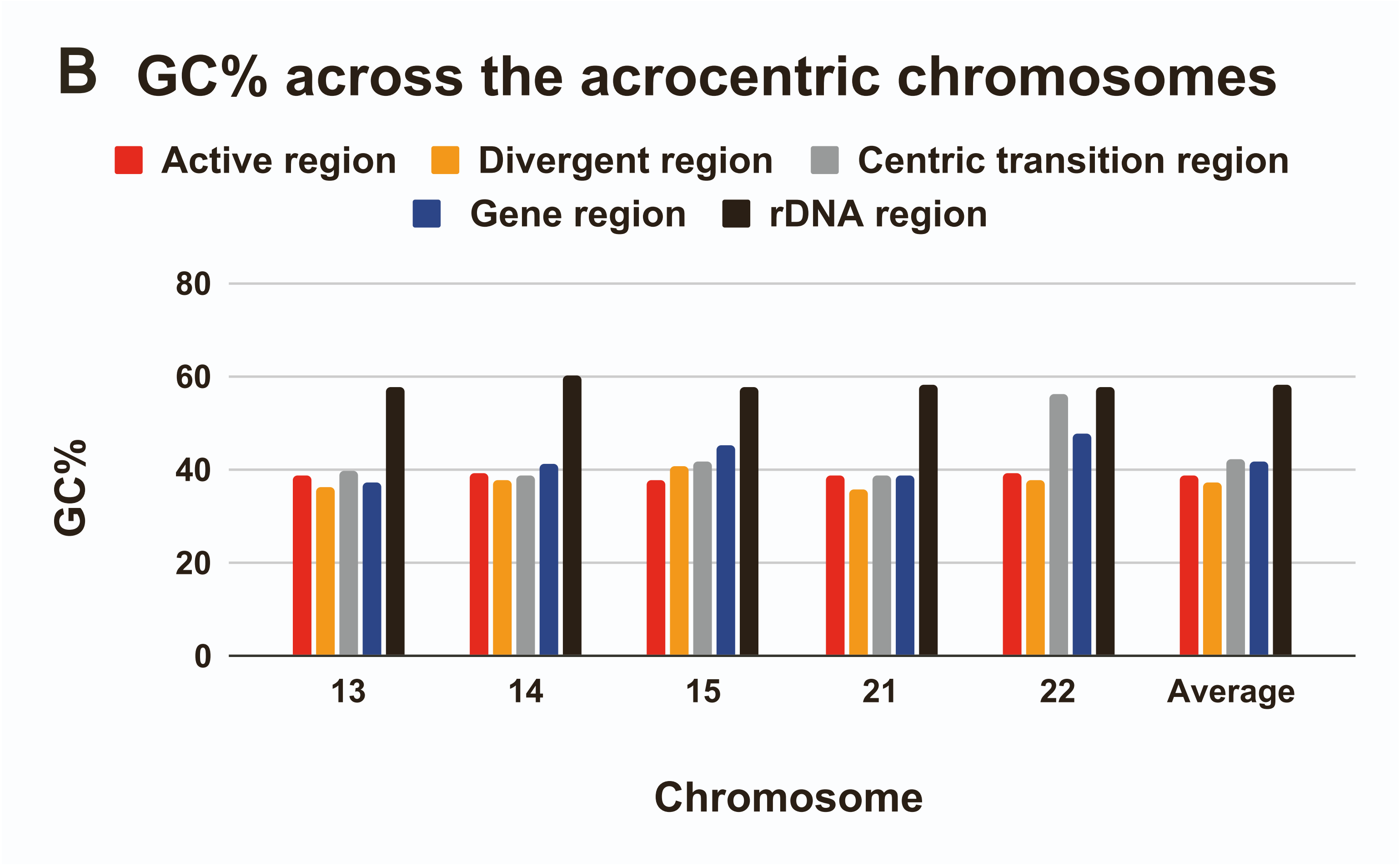
B
GC% across the acrocentric chromosomes
Active region
Divergent region
Centric transition region
Gene region
rDNA region
80
60
40
20
0
GC%
13
14
15
21
22
Average
Chromosome

FIGURE S4

Figure S4. Acrocentric chromosomes comparison. (A) Occupancy of repeat motifs, namely, Direct repeats, G4 motifs, Inverted repeats, Mirror repeats, Z-DNA motifs and Short tandem repeats in the rDNA region. (B) Comparison of GC-content in the rDNA region against the peri/centromeric regions as well as the gene region.

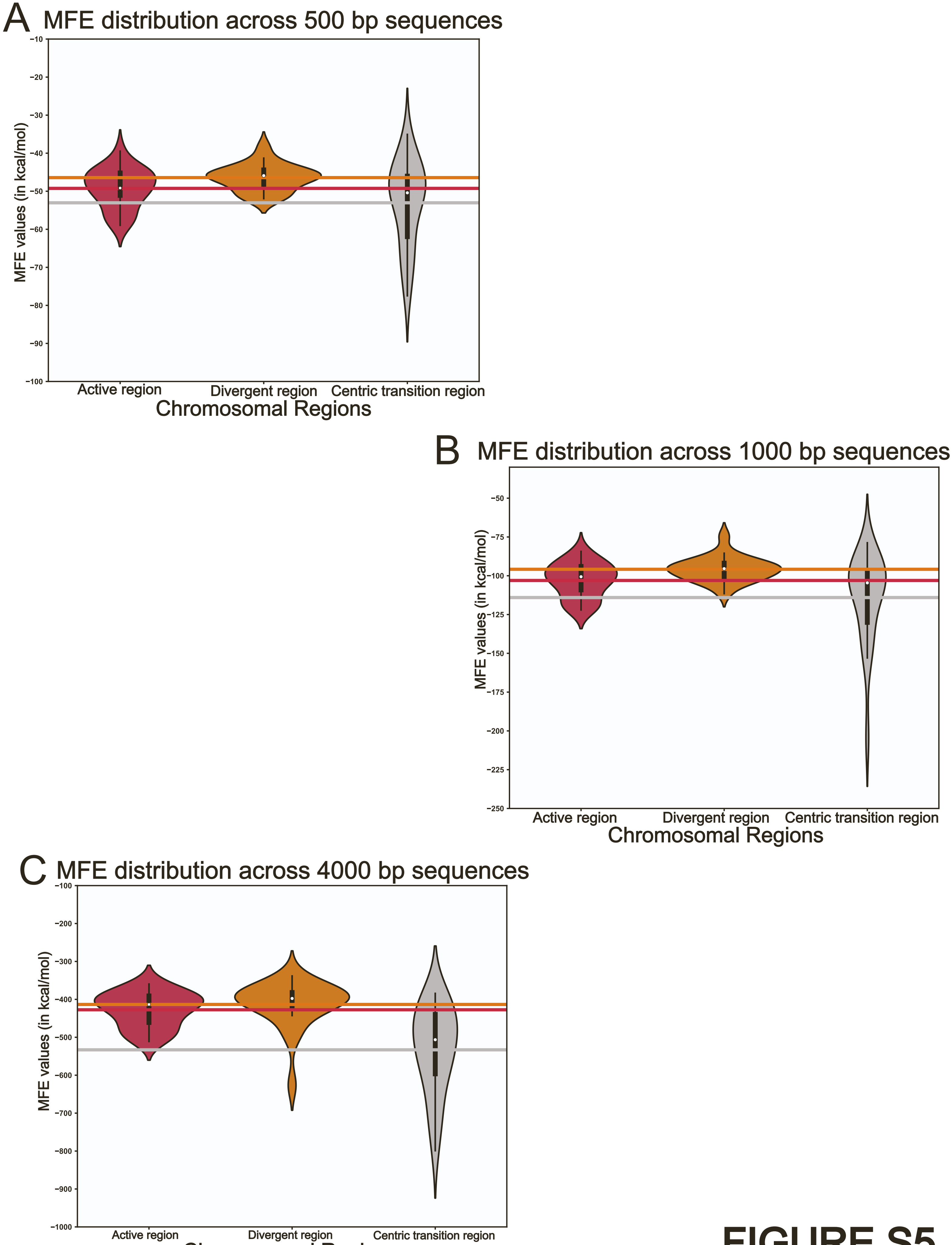
A
MFE distribution across 500 bp sequences
MFE values (in kcal/mol)
−10
−20
−30
−40
−50
−60
−70
−80
−90
−100
Active region
Divergent region
Centric transition region
Chromosomal Regions
B
MFE distribution across 1000 bp sequences
MFE values (in kcal/mol)
−50
−75
−100
−125
−150
−175
−200
−225
−250
Active region
Divergent region
Centric transition region
Chromosomal Regions
C
MFE distribution across 4000 bp sequences
MFE values (in kcal/mol)
−100
−200
−300
−400
−500
−600
−700
−800
−900
−1000
Active region
Divergent region
Centric transition region
Chromosomal Regions

FIGURE S5

Figure S5. MFE value distribution across centromeric sequences of varying lengths. (A) A violin plot displaying the spread of minimum free energy values in the 500bp sequences in the active region, the divergent region and the centric transition region. The horizontal lines correspond to the mean MFE value in each region. (B) A violin plot displaying the spread of minimum free energy values in the 1000bp sequences in the active region, the divergent region and the centric transition region. The horizontal lines correspond to the mean MFE value in each region. (C) A violin plot displaying the spread of minimum free energy values in the 4000bp sequences in the active region, the divergent region and the centric transition region. The horizontal lines correspond to the mean MFE value in each region.

A RPE-1 HAPLOTYPE 1: |MFE| VS PROBABILITY OF MISSEGREGATION

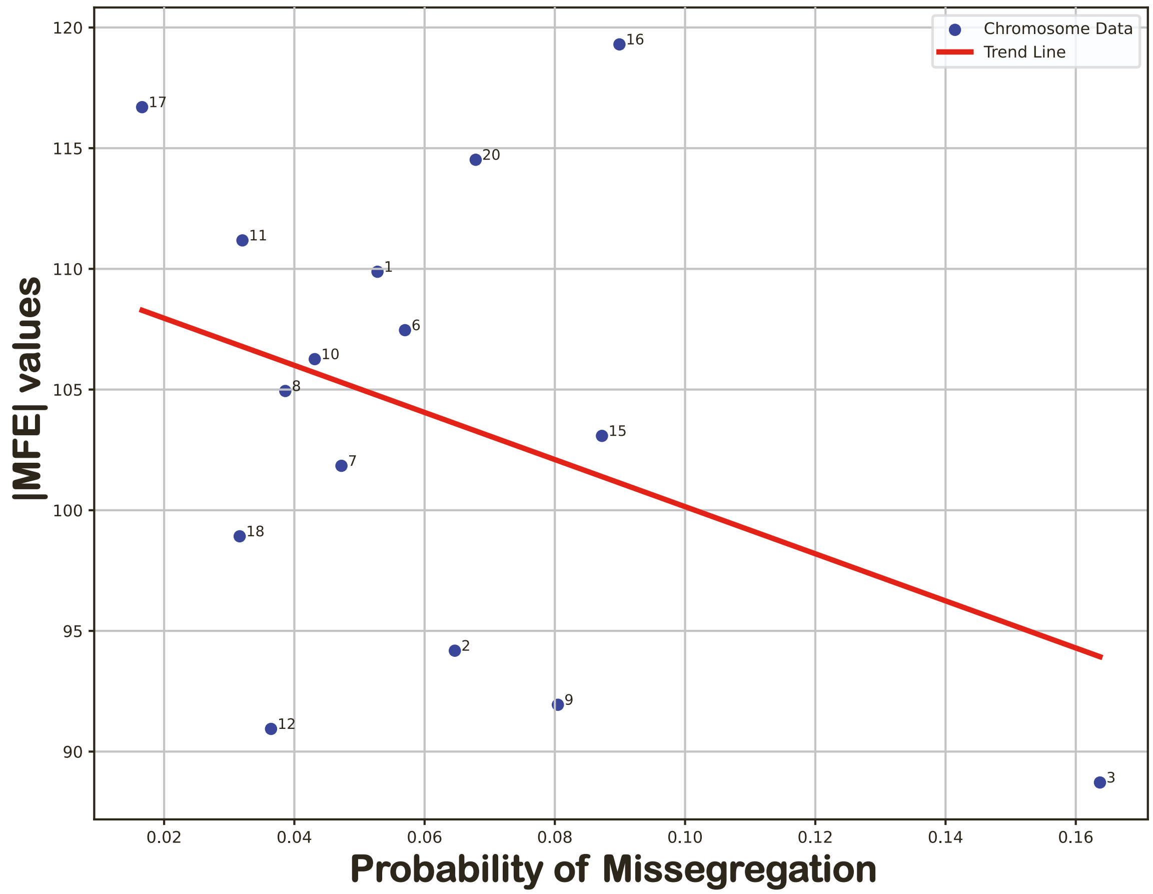

B RPE-1 HAPLOTYPE 2: |MFE| VS PROBABILITY OF MISSEGREGATION

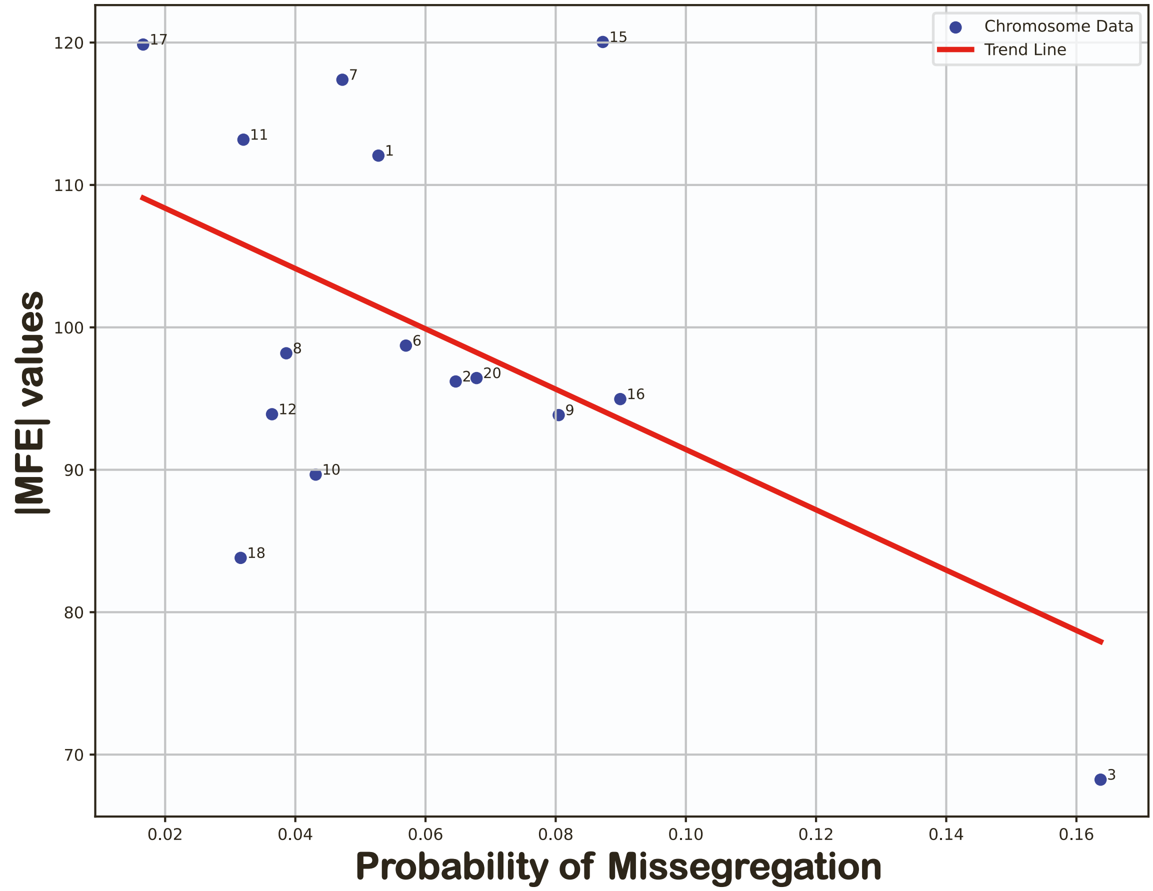

FIGURE S6

Figure S6. Correlation between the |MFE| values and the probability of missegregation. (A) Scatterplot showing the probability of missegregation on the x-axis and the |MFE| values of the secondary structures predicted from sequences extracted from the active region of RPE1 haplotype 1 on the y-axis. A correlation of -0.36 was found between the values on the x-axis and the y-axis. (B) Scatterplot showing the probability of missegregation on the x-axis and the |MFE| values of the secondary structures predicted from sequences extracted from the active region of RPE1 haplotype 2 on the y-axis. A correlation of -0.52 was found between the values on the x-axis and the y-axis. The missegregation probability values were obtained from [1].

Tables summary

Table S1 – Sequences and derived data evaluated in the study for linear DNA of 4000 bp.

Tabs:

1. Sequence Data: DNA sequences extracted and utilized in this analysis along with their genomic coordinates and their region information.
2. Thermodynamic Data: thermodynamic stability data, namely, the Minimum free energy values, the free energy of the thermodynamic ensemble values and the ensemble diversity values of the secondary structures predicted from the DNA sequences present in Sequence Data spreadsheet. This data was obtained using the tool RNAfold [2].
3. Ensemble Diversity Pivot Table: Ensemble Diversity data pivot table displaying the average ensemble diversity values of DNA sequences from all the chromosomes considered across the regions under study. This data was obtained using the tool RNAfold [2].
4. FETE Pivot Table: Free energy of the Thermodynamic Ensemble data pivot table displaying the average free energy of the thermodynamic ensemble values of DNA sequences from all the chromosomes considered across the regions under study. This data was obtained using the tool RNAfold [2].
5. Probability_of_missegregation: Spreadsheet containing the information regarding the probability of missegregation across multiple chromosomes. This data was obtained from [1].
6. MFE Pivot Table: Minimum free energy data pivot table displaying the average minimum free energy values of DNA sequences from all the chromosomes considered across the regions under study. This data was obtained using the tool RNAfold [2].

Table S2 – Non-B DNA output data evaluated in the study.

Tabs:

1. Active region: information on the various non-B DNA motifs detected in the DNA sequences in the active HOR region. This information was obtained using the tool Non-B DNA search tool (nBDST) [3]
2. Divergent region: information on the various non-B DNA motifs detected in the DNA sequences in the divergent HOR region. This information was obtained using the tool Non-B DNA search tool (nBDST) [3]
3. Centric Transition region: information on the various non-B DNA motifs detected in the DNA sequences in the centric transition region. This information was obtained using the tool Non-B DNA search tool (nBDST) [3]
4. rDNA region: information on the various non-B DNA motifs detected in the DNA sequences in the rDNA region. This information was obtained using the tool Non-B DNA search tool (nBDST) [3]

Table S3 – Dyad density and symmetry evaluated in the study.

Tabs:

1. Dyad Symmetries: density of dyad symmetries found in the peri/centromeric regions, namely, the active HOR, the divergent HOR and the centric transition region. This data was obtained using the tool Palindrome by EMBOSS [4].
2. Dyad Symmetries Pivot Table: pivot table displaying the average dyad density in the peri/centromeric regions, namely, the active HOR, the divergent HOR and the centric transition region in the chromosomes considered for this study. This data was obtained using the tool Palindrome by EMBOSS [4].

Table S4 – Sequences and GC content evaluated in the study.

Tabs:

1. GC%: the genomic coordinates of the DNA sequences extracted along with the region information, sequence information and the GC%.
2. GC% Pivot Table: pivot table containing the average GC% values observed in the DNA sequences extracted from various chromosomes across the various genomic regions considered in our study.

<u>Table S5 – Sequences and data derived and evaluated in the study for linear DNA of 500 and 1000 bp.</u>

Tabs:

1. CHM13 500bp sequences: the genomic coordinates of the 500bp sequences extracted along with their chromosome, region and sequence information. This spreadsheet also contains thermodynamic stability data, namely, the minimum free energy (MFE) values, free energy of the thermodynamic ensemble (FETE) values and ensemble diversity (ED) values pertaining to the secondary structures predicted to form by these DNA sequences. The thermodynamic stability data was obtained using the tool RNAFold [2].
2. CHM13 500bp sequences MFE Pivot: pivot table containing the average MFE values of the 500bp sequences extracted from the peri/centromeric regions, namely, the active HOR region, the divergent HOR region and the centric transition region. The thermodynamic stability data was obtained using the tool RNAFold [2].
3. CHM13 1000bp sequences: the genomic coordinates of the 1000bp sequences extracted along with their chromosome, region and sequence information. This spreadsheet also contains thermodynamic stability data, namely, the minimum free energy (MFE) values, free energy of the thermodynamic ensemble (FETE) values and ensemble diversity (ED) values pertaining to the secondary structures predicted to form by these DNA sequences. The thermodynamic stability data was obtained using the tool RNAFold [2].
4. CHM13 1000bp sequences MFE Pivot: pivot table containing the average MFE values of the 500bp sequences extracted from the peri/centromeric regions, namely, the active HOR region, the divergent HOR region and the centric transition region. The thermodynamic stability data was obtained using the tool RNAFold [2].

Table S5 – RPE-1 Chromosome missegregation analyses data.

Tabs:

1. RPE1Hap1_active_region_MFE: This spreadsheet contains the genomic coordinates of the 1000bp long DNA sequences extracted from the active HOR region of the centromere in the Haplotype 1 of the RPE1 genome. This spreadsheet also holds the sequence data and the thermodynamic stability information, namely, the minimum free energy (MFE), free energy of the thermodynamic ensemble (FETE) and ensemble diversity (ED) of the secondary structures these DNA sequences are predicted to form. The thermodynamic stability data was obtained using the tool RNAFold [2].
2. RPE1Hap1 MFE Pivot table: This spreadsheet is a pivot table displaying the average MFE, FETE and ED values of the secondary structures predicted to form by the DNA sequences extracted from the active HOR region of chromosomes considered. The thermodynamic stability data was obtained using the tool RNAFold [2].
3. RPE1Hap1_MFE vs Missegregation: This spreadsheet displays the average MFE values of secondary structures predicted to form from the DNA sequences of the active HOR region in haplotype 1 of the RPE-1 genome next the probability of missegregation information. The data is sorted in ascending order in regard with the probability of missegregation. The missegregation probability information was obtained from [1].
4. RPE1Hap2_active_region_MFE: This spreadsheet contains the genomic coordinates of the 1000bp long DNA sequences extracted from the active HOR region of the centromere in the Haplotype 2 of the RPE1 genome. This spreadsheet also holds the sequence data and the thermodynamic stability information, namely, the minimum free energy (MFE), free energy of the thermodynamic ensemble (FETE) and ensemble diversity (ED) of the secondary structures these DNA sequences are predicted to form. The thermodynamic stability data was obtained using the tool RNAFold [2].
5. RPE1Hap2 MFE Pivot table: This spreadsheet is a pivot table displaying the average MFE, FETE and ED values of the secondary structures predicted to form by the DNA sequences extracted from the active HOR region of chromosomes considered in haplotype 2 of the RPE-1 genome. The thermodynamic stability data was obtained using the tool RNAFold [2].
6. RPE1Hap2_MFE vs Missegregation: This spreadsheet displays the average MFE values of secondary structures predicted to form from the DNA sequences of the active HOR region in haplotype 2 of the RPE-1 genome next the probability of missegregation information. The data is sorted in ascending order in regard with the probability of missegregation. The missegregation probability information was obtained from [1].

References

1. Dumont M, Gamba R, Gestraud P, Klaasen S, Worrall JT, De Vries SG, et al. Human chromosome-specific aneuploidy is influenced by DNA-dependent centromeric features. EMBO J 2020;39:e102924. https://doi.org/10.15252/embj.2019102924.
2. Lorenz R, Bernhart SH, Höner Zu Siederdissen C, Tafer H, Flamm C, Stadler PF, et al. ViennaRNA Package 2.0. Algorithms Mol Biol 2011;6:26. https://doi.org/10.1186/1748-7188-6-26.
3. Cer R, Bruce K, Donohue D, Temiz N, Mudunuri U, Yi M, et al. Searching for non-B DNA-forming motifs using nBMST (non-B DNA Motif Search Tool). Curr Protoc Hum Genet 2012;CHAPTER:Unit-18.722. https://doi.org/10.1002/0471142905.hg1807s73.
4. [36] Rice P, Longden I, Bleasby A. EMBOSS: the European Molecular Biology Open Software Suite. Trends Genet 2000;16:276–7. https://doi.org/10.1016/s0168-9525(00)02024-2.
